# miRNA profiles in livers with different mass deficits after partial hepatectomy and miR-106b~25 cluster accelerating hepatocyte proliferation in rats

**DOI:** 10.1038/srep31267

**Published:** 2016-08-10

**Authors:** Xiao Xu, Zhikun Liu, Jianguo Wang, Qi Ling, Haiyang Xie, Haijun Guo, Xuyong Wei, Lin Zhou, Shusen Zheng

**Affiliations:** 1 Division of Hepatobiliary and Pancreatic Surgery, First Affiliated Hospital, Zhejiang University School of Medicine, Hangzhou, China; 2 Key Lab of Combined Multi-Organ Transplantation, Ministry of Public Health, China

## Abstract

Partial hepatectomy (PH) promotes the reentry of quiescent hepatocytes into cell cycle for regrowth. miRNA profiles in livers with different mass deficits after PH have not been investigated and miRNAs implicated in liver regeneration remain unclear. We generated miRNA profiles from normal and remnant livers at 6, 12, 24, and 36 hours after 1/3 or 2/3PH using microarrays. Compared with normal livers, the proportion of altered miRNAs decreased with time after 1/3PH, but increased after 2/3PH. Most of altered miRNAs between 1/3 and 2/3PH exhibited similar up- or down-regulation, but lower expression magnitude for 1/3PH. Among differentially expressed miRNAs between 2/3PH with robust DNA replication and 1/3PH with a minimal replicative response, we identified miR-101a, miR-92a, miR-25, miR-93 and miR-106b as key regulators of cell cycle. In 2/3PH model, overexpression of miR-106b~25 cluster tended to accelerate liver regeneration, while inhibition of miR-106b~25 cluster markedly repressed regenerative response and delayed recovery of liver function. Mechanistically, RB1 and KAT2B with cell cycle arrest activity were identified as novel targets of miR-106b/93 and miR-25, respectively. Overall, we featured miRNA profiles and dynamics after 1/3 and 2/3PH, and identified miR-106b~25 cluster as being involved in timely cell cycle entry of hepatocytes after PH.

Partial hepatectomy (PH) is commonly performed to treat hepatic tumors. After PH, the lost hepatic mass is restored by liver regeneration, during which quiescent hepatocytes reenter the cell cycle^1^. Liver regeneration is also observed in grafts of living donor liver transplantation and in the remnant liver after living donation^2^. In the rat, the remnant liver can recover its original mass and function within 7–10 days after PH^3^,^4^. Liver regeneration following PH is a very complex but well-orchestrated phenomenon, and many genes participate in the process^5^,^6^. The process begins with priming hepatocytes to enter the cell cycle and undergo one or two rounds of synchronous DNA replication followed by mitosis, and then return to a quiescent state^7^. DNA synthesis in hepatocytes begins at 12 hours and peaks at 24 hours after 2/3 PH in the rat^8^. However, the physiological role of this initiation period and its underlying mechanisms remain under investigation.

It has become evident that posttranscriptional regulation of gene expression is a central component of the cellular gene regulatory network. miRNAs are the most abundant class of small endogenous noncoding 21- to 23-nucleotide RNAs, and they can bind to the 3′ untranslated regions (3′ UTR) of mRNAs to form the RNA-induced silencing complex, where further regulation occurs^9^. miRNAs are involved in many biological processes, such as tumorigenesis^10^,^11^, stem cell differentiation^12^,^13^ and immune responses^14^,^15^. It has been reported that miR-221 promote liver regeneration by targeting Arnt^16^. miRNAs play a pivotal role in promoting the growth of small-size grafts and remaining livers^17^. Several important questions that have not yet been explored include: 1) the relationship between miRNA profiles and deficits in liver mass after PH and 2) the extent to which the widespread changes in miRNA expression that occur after 2/3 PH are linked to hepatocyte DNA replication and liver regeneration. To answer the second query is difficult for various reasons, for instance, because of the confounding factors created by surgical stress and the difficulties in choosing adequate controls for 2/3PH.

To address these questions, we compared the miRNA expression profile after 2/3 PH, a standard procedure that leads to robust DNA synthesis, with that after 1/3 PH, a procedure that causes minimal replication. The patterns and dynamics of miRNA profiles after PH were featured, providing a rich resource of miRNAs underling mechanisms of liver regeneration. Next we focused on miRNA alterations that significantly differed between 1/3 and 2/3 PH during the peak of DNA synthesis. We showed that miR-101a, miR-92a, miR-25, miR-93 and miR-106b were associated with the cell cycle *in vivo* and that the miR-106b~25 cluster is essential for the timely cell cycle reentry of hepatocytes after PH by targeting RB1 and KAT2B.

## Results

### miRNA profiles during liver regeneration after 1/3 and 2/3 PH

**Figure 1:**
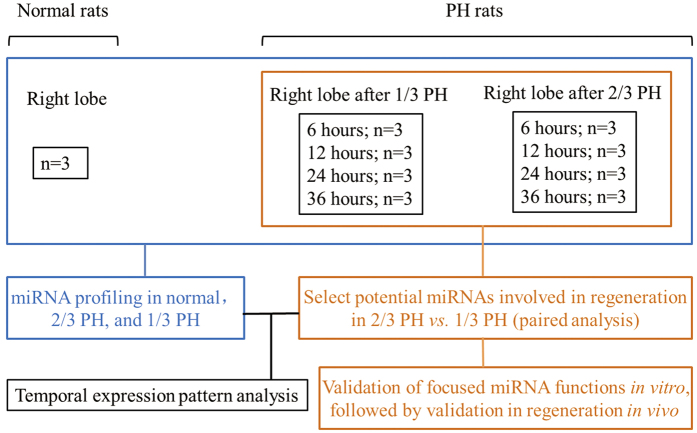
Experimental design. The right hepatic lobes of normal rats were harvested as the normal controls (n = 3). The 1/3 and 2/3 PH surgeries were performed (3 rats at each type of surgery at each time point) and the remnant livers (right lobes) were harvested at 6, 12, 24, and 36 hours post-PH.

**Figure 2:**
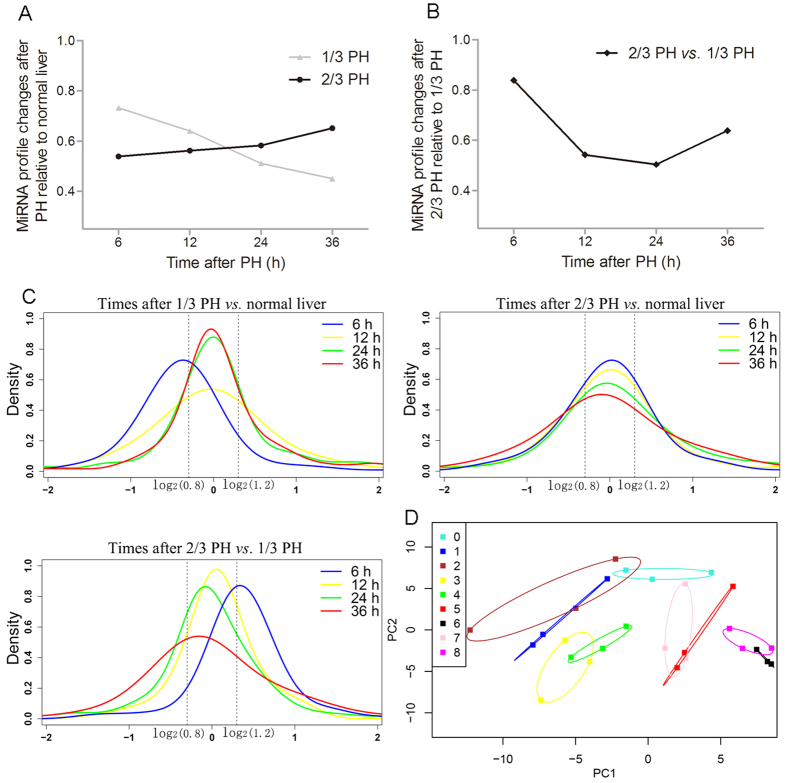
Similarities and differences in miRNA expression patterns after 1/3 and 2/3 PH. (**A**) The number of differentially expressed miRNAs after 1/3 and 2/3 PH compared to normal liver controls. (**B**) The number of preferentially expressed miRNAs after 2/3 PH compared to 1/3 PH. (**C**) Densities representing the distribution of all miRNAs at 6 (blue), 12 (yellow), 24 (green), and 36 hours (red) after PH; the dotted line indicates the threshold of a 1.2 fold-change. The distribution of miRNA changes at 6, 12, 24, and 36 hours showed a shift in 1/3 PH *vs* normal control and 2/3 PH *vs* 1/3 PH. (**D**) Principal component analysis of all investigated samples showed that 1/3 PH induced a fluctuation with a trend to close to that of normal liver over time, while 2/3 PH diverged away from normal liver. 0, normal liver; 1, 3, 5, and 7 represent 6, 12, 24, 36 and hours after 1/3PH, respectively; 2, 4, 6, and 8 represent 6, 12, 24, and 36 hours after 2/3PH, respectively.

We profiled miRNAs in remnant livers from 6 to 36 hours after 1/3 and 2/3 PH, and using normal livers as a control (Fig. 1). All of the detected miRNAs are shown in Supplementary Table 1. The microarray results were confirmed by measuring the expression levels of 9 miRNAs (3 random miRNAs from each expression pattern, namely up-regulated, unchanged, and down-regulated) using real-time PCR, and a strong correlation between the two measurements was observed (Supplementary Figure 1). First, we investigated the expression patterns of miRNAs at 6, 12, 24, and 36 hours after 1/3 and 2/3 PH in comparison to normal livers. Based on their expression levels, the miRNAs were grouped into three sets: down-regulated (<0.8-fold), unchanged (0.8- to 1.2-fold), and up-regulated (>1.2-fold). The proportion of differentially expressed miRNAs (<0.8 or >1.2-fold) to all detected miRNAs decreased from 73% at 6 hours to 45% at 36 hours after 1/3 PH. After 2/3 PH this proportion increased from 54% at 6 hours to 65% at 36 hours after 2/3 PH (Fig. 2A). Next, we selected miRNAs that were preferentially expressed after 2/3 PH compared with 1/3 PH using the same criteria described above (<0.8 or >1.2-fold). The proportion decreased from 84% at 6 hours to 54% at 12 hours and 50% at 24 hours, and it then increased to 63% at 36 hours (Fig. 2B). The similar trend was also observed in Fig. 2C, the overall distribution of temporal miRNA patterns (up-regulated, no change, down-regulated) after PH varies when subjected to different comparisons. For example, at 6 hours after PH, more miRNAs are down-regulated in 1/3 PH vs normal control, while more miRNAs are up-regulated in 2/3 PH vs 1/3 PH. Principal component analysis (PCA) allows large datasets to be devolved into a few constructs that contain most of the variance. PCA of all of the investigated samples revealed distinct trends for the miRNA profilesafter 1/3 and 2/3 PH (Fig. 2D). Specifically, 1/3 PH induces a fluctuation around the normal liver profile and returns to a near-normal liver profile within 36 hours, while 2/3 PH cause a divergence from the normal liver profile as the liver prepares for obvious hepatocyte proliferation.

**Figure 3:**
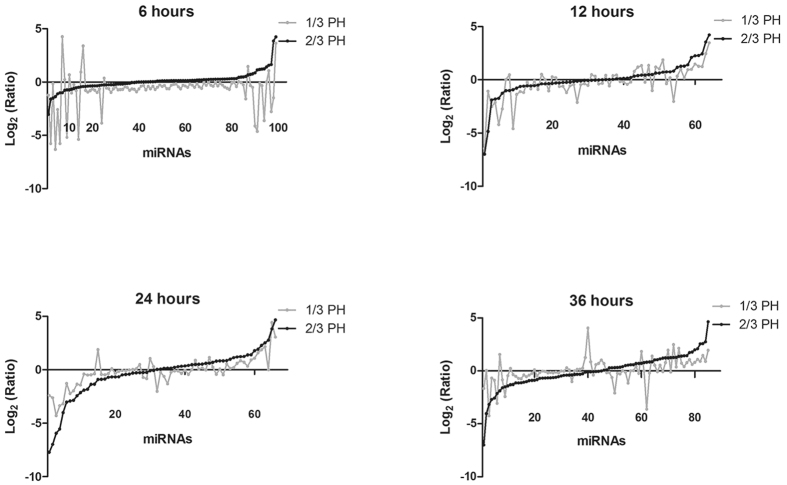
Similarities and differences in the miRNA expression patterns between 1/3 and 2/3 PH. The Log_2_ ratios of preferentially expressed miRNAs after 1/3 (gray line) and 2/3 PH (black line) relative to normal controls. First, miRNAs that are preferentially expressed after 2/3 PH relative to 1/3 PH were selected by fold change (<0.8-fold or >1.2-fold). The selected miRNAs were sorted by the Log_2_ (Ratio) values (2/3 PH relative to normal). Next the Log_2_ (Ratio) values of the same miRNAs after 1/3 PH were plotted. The data showed that up- or down-regulation of miRNA expression is of greater magnitude after 2/3 PH except at 6-hour time point.

**Figure 4:**
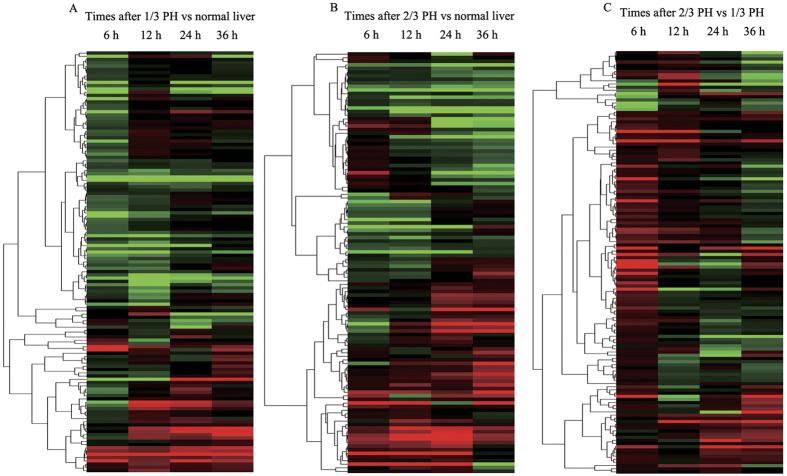
Heat maps of miRNA profiles. Heat map of miRNA changes after (**A**) 1/3 PH or (**B**) 2/3 PH samples relative to normal liver and (**C**) in 2/3 PH *vs*. 1/3 PH at the 4 different time points after PH. Increases in miRNA levels are indicated by red, green denotes a decrease, and no change in miRNA levels are indicated with black.

To learn more about the differences between 1/3 and 2/3 PH, we plotted the log2 ratios of the preferentially expressed miRNAs in the regenerating liver relative to the normal liver at each time point after 1/3 or 2/3 PH (Fig. 3). The data showed that, except at 6 hours after PH, the magnitude of the expression of most miRNAs was greater, whether up or down, after 2/3 PH than 1/3 PH. To identify common regulatory responses between the different types of surgery, we clustered the miRNAs into Venn diagrams according to their type of regulation, combined with time after PH (Supplementary Figure 2). Except for the 6-hour time point, more than half of the altered miRNAs changed in the same direction. These data suggest that 1/3 and 2/3 PH share common miRNAs whose expression changes with a similar regulatory regulation direction but with a reduced magnitude in 1/3 PH. To more clearly elucidate the temporal pattern of miRNA changes, the miRNAs with expression level changes of up to 1.2-fold in at least one of the time points were selected to generate a heat map (Fig. 4). Venn diagrams were draw according to regulatory direction (up/down), combined with the type of surgery (Supplementary Figure 3). These results showed that the miRNA levels undergo dynamic changes during liver regeneration after PH and clearly display a clear biphasic expression pattern, reflecting their key roles in regulating the regenerative process.

Overall, these results reveal the different dynamics of miRNA profiles after 1/3 and 2/3PH and indicate that the large miRNA response in the first 6 hours after PH does not predict whether DNA replication will occur.

### miRNAs involved in liver regeneration after PH

**Figure 5:**
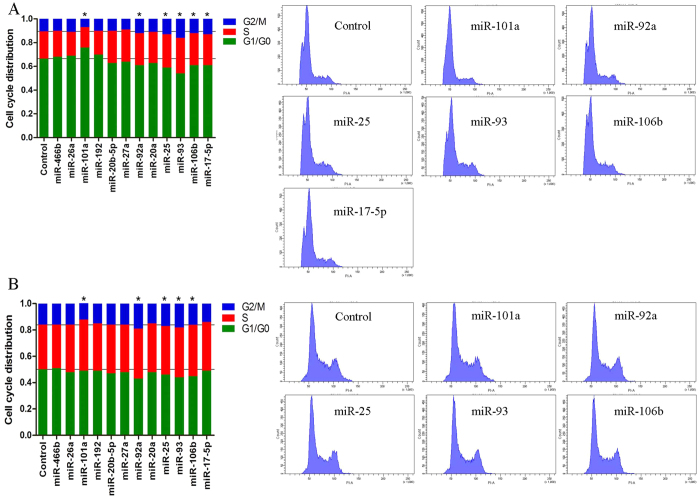
Cell cycle profiles affected by miRNAs. The cell cycle distribution was determined by flow cytometry in BRL (**A**) and RH-35 (**B**) cells transfected with the negative control or with candidate miRNA mimics for 48 hours. The data for all miRNAs were expressed as the mean of 3 independent experiments, which are shown in the left panels. Grid lines represent the cell cycle distribution of the control. *P < 0.05 compared with the negative control. Representative figures for miRNAs that had a significant effect on the cell cycle distribution are shown in the right panels.

To identify the specific miRNAs involved in liver regeneration, we needed to select the appropriate controls by controlling the confounding factors induced by surgical stress. It is well known that 2/3 PH induces marked liver regeneration and robust DNA replication peaks at 24 hours after PH^8^. The ideal control would be an operation that also causes liver mass loss but does not lead to marked liver regeneration. In our model, 1/3 PH causes minimal replication (Supplementary Figure 4). Thus, we chose 1/3 PH as a control for 2/3 PH to query for miRNAs involved in liver regeneration. This strategy differs from previous publications using a sham operation as the control. We selected 12 unique miRNAs that were significantly up- or down- regulated at the 24-hour timepoint after 2/3 PH *vs*. 1/3 PH (*P* < 0.05). We analyzed the cell cycle distributions of cells after transfecting rat cell lines (RBL, RH-35) with these miRNAs. We found that 6 miRNAs significantly affected the cell cycle in BRL cells and 5 significantly affected RH-35 cells; the cell lines shared 5 common miRNAs (Fig. 5). In details, miR-101a was down-regulated, and it decreased cell number in the G2-M phase with consistently increases in cell number in the G1 or S phases; meanwhile, miR-92a, miR-25, miR-93, and miR-106b were up-regulated, and they decreased the G1 cell population while concomitantly increasing the accumulation of cells in the S and G2 phases. No significant results were observed in an analysis of cell apoptosis after transfection with these miRNAs (data not shown). Furthermore, the expression patterns of these functional miRNAs were confirmed in normal liver and the remnant liver at serial times after 1/3 and 2/3 PH by real time-PCR.As we expected, miR-106b, miR-93, miR-25 and miR-92a were significantly up-regulated at 24 and 36 hours after 2/3 PH, and miR-101a was down-regulated at 12, 24 and 36 hours after 2/3 PH (Supplementary Figure 5).

### The miR-106b~25 cluster accelerates liver regeneration after PH

**Figure 6:**
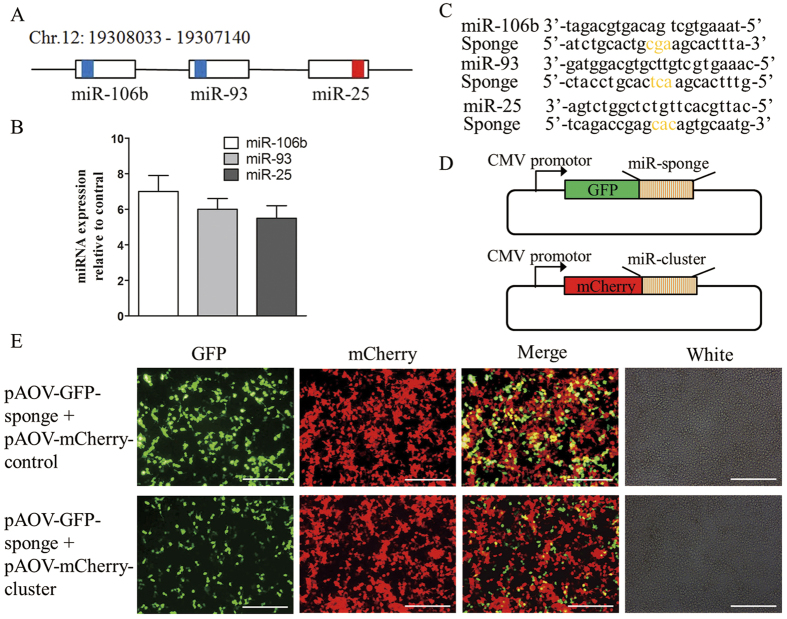
Generation of the AAV9-miR-106b~25 cluster or sponge and verification. (**A**) Schematic illustration of the miR-106b~25 cluster and the positions of the constituent miRNAs in the genome. The boxes represent the miRNA precursor sequences, with the colored sections showing the mature sequences, miRNAs sharing the same seed sequences are shaded with the same color. Chr, chromosome. (**B**) Real-time PCR revealed that BRL cells transfected with the AAV9-miR-106b~25 vector expressed much more miR-106b, miR-93 and miR-25 than did the cluster control vector after 2 days. (**C**) Sequences of miRNAs and sponges. The yellow indicates bulges in the sponge sequence. Base pair mismatches minimize the enzymatic degradation of the sponge-miRNA pair. (**D**) The pAOV-GFP-sponge vector contained the GFP gene and a 3′ UTR which consisted of 6 repeated sponges for miR-25, miR-93 and miR-106b, while the pAOV-mCherry-cluster vector contained the mCherry gene and the precursor sequences of miR-25, miR-93 and miR-106b. (**E**) In 293 T cells cotransfected with pAOV-GFP-sponges and pAOV-mCherry-cluster or pAOV-mCherry-control for 48 hours, the intensity of the GFP produced from the vector with the sponges decreased along with overexpression of the miR-106b~25 cluster compared to the control. Scale bar = 200 μm; original magnification 100×, Olympus.

As demonstrated above, miR-92a, miR-25, miR-93, and miR-106b are associated with faster entry into the S and/or G2 phases. Interestingly, miR-25, miR-93, and miR-106b are members of the miR-106b~25 cluster located within about 0.5 kb on chromosome 12 (Fig. 6A). The miR-106b~25 cluster is reportedly overexpressed in human cancer, and it acts as an oncogene by targeting p21 and Bim^18^,^19^. In addition, its members modulate cell proliferation and differentiation and angiogenesis^20^,^21^. These findings led us to further pursue the role of the miR-106b~25 cluster in liver regeneration. Systemic administration of adeno-associated virus (AAV) vectors results in long-term, robust transgene expression with minimal toxicity and immune responses in several animal models^22^. Gain- or loss-of function studies were performed in the rat liver using AAV9 vectors expressing the miR-106b~25 cluster or sponges for this cluster, respectively. BRL cells transfected with a vector harboring the AAV9-miR-106b~25 cluster expressed more miR-25, miR-93, and miR-106b than cells transfected with the AAV9 cluster control vector, as assessed using real-time PCR (Fig. 6B). AAV9-miR-106b~25 sponges are transcripts with 6 repeated miRNA antisense sequences of miR-25, miR-93 and miR-106b (Fig. 6C). To assess whether the AAV9-miR-106b~25 sponge vectors were functional, we used an expression vector containing a gene encoding green fluorescent protein (GFP) with the miR-106b~25 sponge sequence added in the 3′ UTR of the gene encoding GFP (pAOV-GFP-sponges; Fig. 6D) and confirmed the effective binding of the miRNAs to the sponges. The miR-106b~25 cluster vector (pAOV-mCherry-cluster, Fig. 6D) expressed mCherry, miR-25, miR-93 and miR-106b. In 293 T cells cotransfected with pAOV-GFP-sponges and pAOV-mCherry-cluster or pAOV-mCherry-control, the intensity of the GFP with the sponges decreased relative to the control with overexpression of the miR-106b~25 cluster (Fig. 6E).

**Figure 7:**
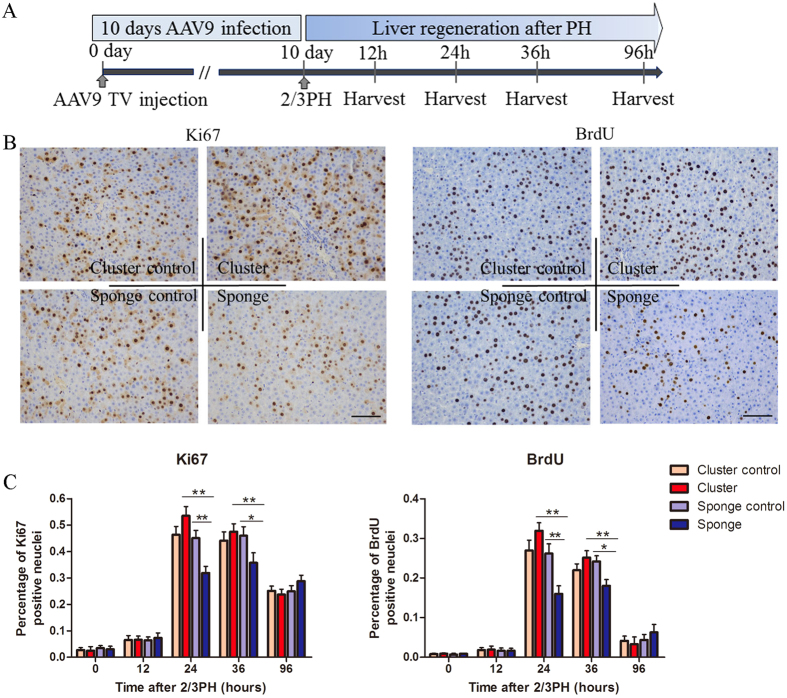
miR-106b~25 cluster association with liver regeneration after 2/3 PH. (**A**) Schematic representation of the experimental design. (**B**) Immunohistochemistry for Ki67 and BrdU showed that rats injected with the AAV9- miR-106b~25 cluster trended toward higher hepatocyte proliferation than rats injected with the AAV9-miR-cluster control, and exhibited significantly lower hepatocyte proliferation in rats with the AAV9-miR-sponge than rats with the AAV9-miR-sponge control. Scale bar = 100 μm; original magnification 200×, Olympus. (**C**) Quantification of Ki67, and BrdU-positive cells at 0, 12, 36, and 96 hours after 2/3 PH. The results represent findings obtained from 5 rats. Error bars represent the SEM. *P < 0.05 and **P < 0.005.

Next, 2 × 10^12^ viral particles of AAV9-miR-106b~25, AAV9-miR-sponge, and their controls were separately injected into rats via the tail vein (TV) (Fig. 7A), AAV9-miR-106b~25, AAV9-miR-sponge, and their controls are hereafter referred to as miR-cluster, miR-sponge, cluster-control and sponge-control, respectively. Ten days after TV injection, we performed 2/3 PH to induce liver regrowth. The expression of miR-106b~25 was analyzed in the livers of rats that were injected with miR-cluster using real time-PCR. We found that miR-106b, miR-93, and miR-25 levels remained higher after PH in the livers of rats injected with miR-cluster than in the livers of control rats; and miR-106b, miR-93 and miR-25 were significantly lower in the miR-sponge-treated rats than that in the controls, especially at 24h and 36 h after 2/3PH (Supplementary Figure 6). In control rats, we found that miR-106b, miR-93, and miR-25 levels also increased after 2/3 PH (Supplementary Figure 6). Liver regeneration was analyzed using Ki67 and BrdU staining. First, we selected the time point of 24 hours after 2/3 PH, which coincided with the peak of DNA synthesis. We found that rats injected with the miR-cluster showed a trend toward higher numbers of Ki67 and BrdU-positive nuclei in hepatocytes compared to their respective controls (P = 0.09 and 0.07, respectively). At the same time, rats injected with the miR-sponge exhibited significantly lower numbers of Ki67-, and BrdU-positive nuclei in hepatocytes at this timepoint (Fig. 7B). In addition, we also analyzed proliferation at 12 36, and 96 hours after 2/3 PH (Fig. 7C). As at 24 hours, we found a significant decrease in proliferation in rats injected with the miR-sponge at 36 hours after 2/3 PH. And miR-sponge-treated rats showed a significant decrease in proliferation than miR-1cluster-treated ones at 24 and 36 hours after 2/3 PH (Fig. 7C). These results indicated that miR-106b~25 is involved in liver regeneration, which is consistent with results suggesting that members of the miR-106b~25 cluster induce hepatocytes to enter more rapidly into S phase. Notably, we did not find any spontaneous proliferation of hepatocytes in miR-106b~25-overexpressing rats at 0 hours. Serum alanine aminotransferase (ALT), aspartate aminotransferase (AST), and total bilirubin (T.Bil) levels at 96 hours after PH were significantly higher in the miR-sponge group compared to the control group, and there were no significant differences observed among the four groups at other timepoints (Supplementary Figure 7). The liver /body weight ratio was significantly higher in the miR-cluster overexpressing rats than the controls at 36 h after 2/3PH, and this ratio remained lower in the sponge-treated rats than the controls from 24 h to 96 h after 2/3PH (Supplementary Figure 8).

### The miR-106b~25 cluster and its targets

**Figure 8:**
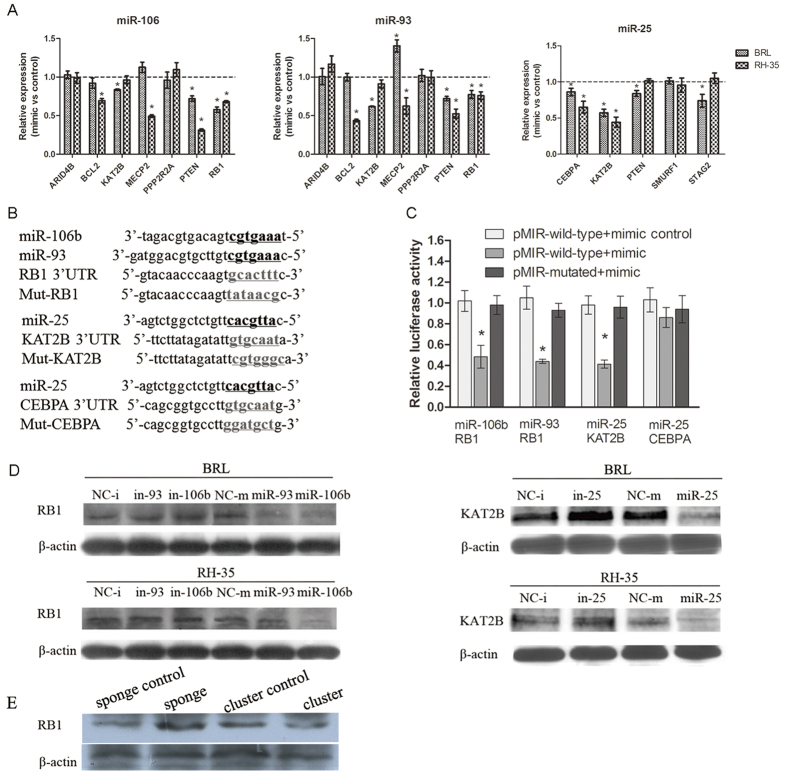
Validation of potential miRNA targets. (**A**) BRL and RH-35 cells were transfected with miR-106b, 93, and 25, and the mimic control was used as a control. After 48 hours, the potential target expression was monitored by real-time PCR and was represented as the expression level relative to the control. The data represent at least 3 independent experiments with SEM. *P < 0.05. (**B**) Schematic view of the miRNA binding sites in the 3′ UTRs of the RB1, KAT2B, and CEBPA genes and their corresponding mutated sites, which were constructed in a luciferase system. (**C**) HEK293T cells were cotransfected with 10 nmol/L miRNA mimics or with the control and different pMIR-constructs. After 48 hours, the cells were harvested and the luciferase activities analyzed. All Renilla luciferase activities were normalized against the firefly luciferase activity. The data represent at least 3 independent experiments with SEM. *P < 0.05. (**D**) miR-106b and miR-93 decreased the protein expression of RB1, and miR-25 decreased KAT2B as assessed by western blot analysis. Western bloting analyses of RB1 and KAT2B in BRL and RH-35 cells transfected miRNA mimics or inhibitors, actin was used as the loading control. (**E**) The representative expression of RB1 in the tissues of animals by western blotting at 24 hours after PH. RB1 was up-regulated by the spong for miR-106~25, and inhibited by miR-106~25.

We used the same samples that were used for the initial miRNA microarrays to perform an additional comparison of the mRNAs that differed between 1/3 and 2/3 PH at 24 hours after PH. Ingenuity Pathway Analysis (IPA) analyses were performed to identify targets of the miR-106b~25 cluster using the microarray data for the total mRNA and for the miRNAs at 24 hours after PH. Potential targets were matched to the 3 miRNAs to produce a target candidate list according to two principles, namely inverse expression and the potential mRNAs to be targets of miRNAs based on *in silico* analyses. Based on our functional data for miR-106b, -93, and -25, which were associated with the cell cycle (Fig. 5), we searched in our target candidate list for targets involved in cell cycle regulation. Several miRNA-mRNA pairs were identified (Supplementary Table 2). A total number of 11 and 16 miRNA–mRNA pairs were identified for miR-25 and miR-93 (miR-106b), respectively. For these focused targets, we first confirmed their expression in BRL and RH-35 cells using real-time PCR (Fig. 8A). The expression levels of RB1 and PTEN were downregulated following miR-106b and miR-93 transfection in both BRL and RH-35 cells, while the levels of CEBPA and KAT2B were downregulated after miR-25 transfection. Of them, PTEN was confirmed as a target of miR-93 and miR-106. Next, we chose RB1, CEBPA, and KAT2B for further confirmation using luciferase reporter assays (Fig. 8B). As shown in Fig. 8C, miR-106b and miR-93 expression significantly reduced the luciferase activities of RB1 reporters by 48% and 44%, respectively, and miR-25 reduced luciferase expression of KAT2B reporters by 41%. This repression was alleviated by mutating the miRNA-binding site in the 3′ UTR of the target gene. However, CEBPA reporter luciferase activities were not repressed by miR-25. The identified targets (RB1 and KAT2B) were further successfully validated by western blot analysis (Fig. 8D). Taken together, these results show that RB1 and KAT2B are targets of miR-106b/93 and miR-25, respectively. The similar result regarding RB1 was observed in the animal at 24 hours after PH (Fig. 8E). But there was no obvious difference about the expression of KAT2B in the livers at 24 hours after PH (data not shown), which could be explained by the more complex environment *in vivo* than that *in vitro*. To query the role of miR106b-25 on cyclins, we further detected the expression of Cdk6, Ccnb1, Ccnb2, Ccne1 and Cdc25 in the tissues of animals at all time-points using real-time PCR. We found that miR-106~25 can affect the expression of Ccnb1 and Cdc25 at 24 hours after PH (Supplementary Figure 9).

## Discussion

To investigate the relationships between miRNA profiles and liver mass deficits after PH and to identify the miRNAs that are linked to hepatocyte DNA replication, we compared the miRNA profiles after 2/3 PH with those after 1/3 PH. The patterns of miRNA expression after 1/3 and 2/3 PH were analyzed and characterized. Next, the differences between 1/3 and 2/3 PH were examined, and potentially important miRNAs were further investigated *in vitro* and *in vivo*. The main findings of this study can be summarized as follows: 1) Despite the lack of marked DNA replication, 1/3 PH also caused widespread changes in miRNA expression, and the number of altered-miRNAs increased after 2/3 PH but decreased after 1/3 PH; 2) More than half of the altered miRNAs were shared between 1/3 and 2/3 PHand changed with similar regulatory direction; 3) The miR-106b~25 cluster is essential for liver regeneration after 2/3 PH and influences the cell cycle by targeting KAT2B and RB1.

In contrast to our initial hypothesis that any miRNA expression changes after 1/3 PH would be relatively small, we found that the changes in miRNA expression after 1/3 PH relative to the normal liver were larger than those after 2/3 PH at 6 hours. At later time points, as we expected, the number of altered miRNAs increased after 2/3 PHbut decreased after 1/3 PH. In the first 6 hours, the liver initially responded to 1/3 PH with even more massive changes in miRNA expression (compared with 2/3 PH), although 1/3 PH did not result in DNA replication. We speculate that 2/3 PH causes excess injury or “shock” to the liver compared with 1/3 PH, and thus the early responses to 2/3 PH may be relatively attenuated. Most of the altered miRNAs during early liver regeneration may be involved in stress responses, nutrition and energy metabolism. At later times, the decision of whether to initiate a replicative response is performed to restore deficits in liver mass. Furthermore, to adequately restore liver mass loss, 2/3 PH requires that more hepatocytes enter the cell cycle than 1/3 PH, and thus the difference relative to normal liver increases after 2/3 PH and decreases after 1/3 PH. We featured the levels of miRNAs after 1/3 and 2/3 PH relative to normal livers and compared 2/3 PH to 1/3 PH.

Specificity toward liver growth and DNA replication seems to occur at or after approximately 12 hours after 2/3 PH, based on the Ki67 and BrdU analysis. The preferentially expressed miRNAs at 24 hours after 2/3 PH compared with 1/3 PH potentially promote the entry of quiescent hepatocytes into the cell cycle for liver growth. Thus, these significantly altered miRNAs were analyzed for their role in the cell cycle; these analyses showed that miR-25, miR-93, and miR~106b were associated with faster entry into the S and G2 phases. The miR-106b~25 cluster consists of these 3 mature miRNAs^23^, which are differentially expressed in human cancers and function as oncogenes in liver tumors^19^,^24^,^25^. As a cluster, miR-106b~25 induces an epithelial-to-mesenchymal transition and a tumor-initiating cell phenotype in human breast cancer by targeting Smad7^26^, and it increases the expression of Snail and enhances cell migration and invasion in non-small cell lung cancer^25^. miR-106b~25 cluster is also essential for neovascularization after hindlimb ischaemia in mice^21^. In the present study, the key role of the miR-106b~25 cluster during liver regeneration was investigated in a rat 2/3 PH model. Inhibition of miR-106b~25 hindered liver regrowth. However, overexpression of the miR-106b~25 cluster revealed a trend toward acceleration of liver growth, despite not reaching statistical significance. This lack of significance may be explained by 2/3 PH itself triggering the upregulation of miR-106b~25, so that endogenously produced miR-106b~25 may mask the effect of exogenous miR-106b~25 overexpression. Additionally, T.Bil plasma concentration at 96 hours after 2/3 PH was significantly higher in the miR-sponge group, reflecting loss of miR-106b~25 could delay recovery of liver function. Furthermore, we identified the targets of miR-106b~25, which contributed to the pro-cell cycle activity of hepatocytes. We identified RB1 and KAT2B as novel targets of miR-106b/93 and miR-25, respectively. RB1 is a negative regulator of the cell cycle and inhibits the G0-G1 transition. It interacts with E2F1 and represses its transcriptional activity, thereby resulting in cell cycle arrest^27^. KAT2B inhibits cell cycle progression and counteracts the mitogenic activity of the adenoviral oncoprotein E1A^28^,^29^. Thus, it is conceivable that up-regulation of the miR106b~25 cluster during PH is vital for the reentry of quiescent hepatocytes into the cell cycle for regrowth. Thus, this study may reveal a crucial molecular mechanism for the miRNA-based modulation of liver growth after PH in rats.

Aside from a growing body of evidence that miRNAs are involved in liver regeneration^30^, there are diverse mechanisms which participate in liver regeneration after PH. These include cytokine signaling, lipid and bile acid metabolism^31^,^32^. miRNAs are reported to effect liver regeneration through the crosstalk with cytokine signaling. Down-regulation of miR-23b contributes to activation of the TGF-b1/Smad3 signalling to terminate liver regeneration^33^. miR-376b is involved in the IL6 signaling transduction system to prime liver regeneration^34^. miRNAs can regulate lipid and bile acid metabolism which are involved in liver regeneration, thus the miRNAs may affect liver regeneration via lipid and bile acid. Our dataset has featured the levels of miRNAs after different mass deficits of liver and firstly used 1/3PH as control for 2/3PH to show the specific miRNAs are crucial to induce liver regeneration. This allows us to find a specific subset of miRNAs that has not been previously identified, such as miR-106b~25 cluster. Similarly, the set of the miRNAs may give indications for direction of further functional studies in liver regeneration.

In summary, this study characterized miRNA profiles during liver regeneration following 1/3 and 2/3 PH. Relative to normal liver, miRNA expression increases after 2/3 PH and decreases after 1/3 PH. Furthermore, 1/3 and 2/3 PH shared many common miRNAs, which changed with a similar regulatory direction under both treatments but with a lower magnitude in 1/3 PH. The different miRNA profiles following 1/3 and 2/3 PH provide a rich resource of miRNAs that may be involved in liver growth. Furthermore, miR-106b~25 promotes liver regeneration after PH, stimulating the rapid S-phase entry of hepatocytes by targeting KAT2B and RB1. Our findings indicate that modulation of miR-106b~25 may represent a new therapeutic approach to treat liver failure caused by hepatocyte injury.

## Materials and Methods

### Surgical Procedures

About 180 g male Sprague-Dawley rats were purchased from the Shanghai Animal Center (China). The left lateral lobe, which accounts for 30% of the total liver mass, was removed for 1/3 PH. In addition, 2/3 PH surgeries were performed as originally described^35^. The regenerating lobes (right lobes) were harvested 6, 12, 24, or 36 hours after surgery (3 rats/time point/type of surgery). The right lobes of 3 rats without PH were also harvested as the normal controls (Fig. 1). To avoid differences related to circadian rhythms in miRNA expression, liver harvest was performed between 8 a.m. and 12 p.m. All experimental procedures involving the animals were carried out according to the guideline of the National Institutes of Health for the care and use of laboratory animals, and the methods were approved by the Animal Care Committee of Zhejiang University.

### miRNA and mRNA microarray expression profiling and validation by real-time PCR

miRNA expression profiles were investigated in normal and remnant livers at the indicated time points using microarrays (Agilent Rat miRNA, 8*15 K, V16.0). Triplicate samples were examined at each time point for miRNA expression profiles using microarrays (Fig. 1), and one array for 1/3PH was excluded from the analysis because of poor quality control. mRNA microarrays (Affymetrix Rat 230 2.0) were only performed for the 24-hour post-PH samples to quickly match potential targets of interesting miRNAs. The results were validated using real-time PCR. For further detail, please refer to the Supplementary Materials and Methods.

### AAV9 preparation

The sequences of the miR-106b~25 cluster and sponges were artificially synthesized. We assumed that a fragment containing not only the miRNA precursor sequence but also tens of flanking bases would result in effective cleavage of the large primary miRNA product into mature miRNAs. The sponges of miR-106b~25 were designed to generate transcripts with repeated miRNA antisense sequences that could sequester miRNAs from their endogenous targets. All constructs were confirmed by sequencing. For more information, please refer to the Supplementary Materials and Methods.

### Cell cycle and apoptosis analysis, immunohistochemical staining and western blotting analysis, luciferase reporter assays and blood laboratory tests

Please refer to the Supplementary Materials and Methods.

## Additional Information

**Accession code:** The microarray data were deposited in GEO (http://www.ncbi.nlm.nih.gov/geo/) with accession number GSE79983.

**How to cite this article**: Xu, X. *et al*. miRNA profiles in livers with different mass deficits after partial hepatectomy and miR-106b~25 cluster accelerating hepatocyte proliferation in rats. *Sci. Rep*. **6**, 31267; doi: 10.1038/srep31267 (2016).

## Supplementary Material

Supplementary Information
