# Supplementary material for: miRNA profiles in livers with different mass deficits after partial hepatectomy and miR-106b~25 cluster accelerating hepatocyte proliferation in rats
Source: Sci Rep. 2016 Aug 10;6:31267. doi: 10.1038/srep31267 (PMC4978973; doi:10.1038/srep31267)
Supplement: Supplementary Information [file srep31267-s1.pdf]

miRNA profiles in livers with different mass deficits after partial hepatectomy and miR-106b~25 cluster accelerating hepatocyte proliferation in rats

Xiao Xu[1 §], MD, PhD, Zhikun Liu[1 §], MD, Jianguo Wang[2, §], MD, Qi Ling[1], Haiyang Xie[2], PhD, Haijun Guo[2], MD, Xuyong Wei[2], MD, PhD, Lin Zhou[2], PhD, and Shusen Zheng[1] * MD, PhD, FACS

[§] These authors contributed equally to this work

*: corresponding author

1. Division of Hepatobiliary and Pancreatic Surgery, First Affiliated Hospital, Zhejiang University School of Medicine, Hangzhou, China

2. Key Lab of Combined Multi-Organ Transplantation, Ministry of Public Health, China

Supplementary materials

Supplementary Figures

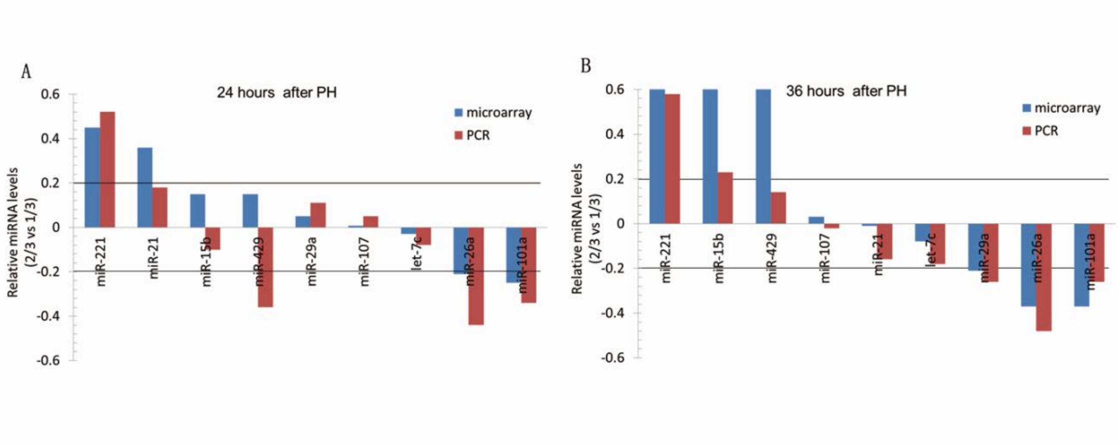

Supplementary Figure 1. Three miRNAs were randomly selected in each expression pattern at 36 hours (up, no-changed, down). Validation of the microarray data was done using real-time PCR on samples of 2/3 *vs.* 1/3PH at 24 and 36 hours after PH. Relative miRNA expression ratios (1/3PH liver considered to be 1) were normalized

against the stably expressed U6 small nucleolar RNA.

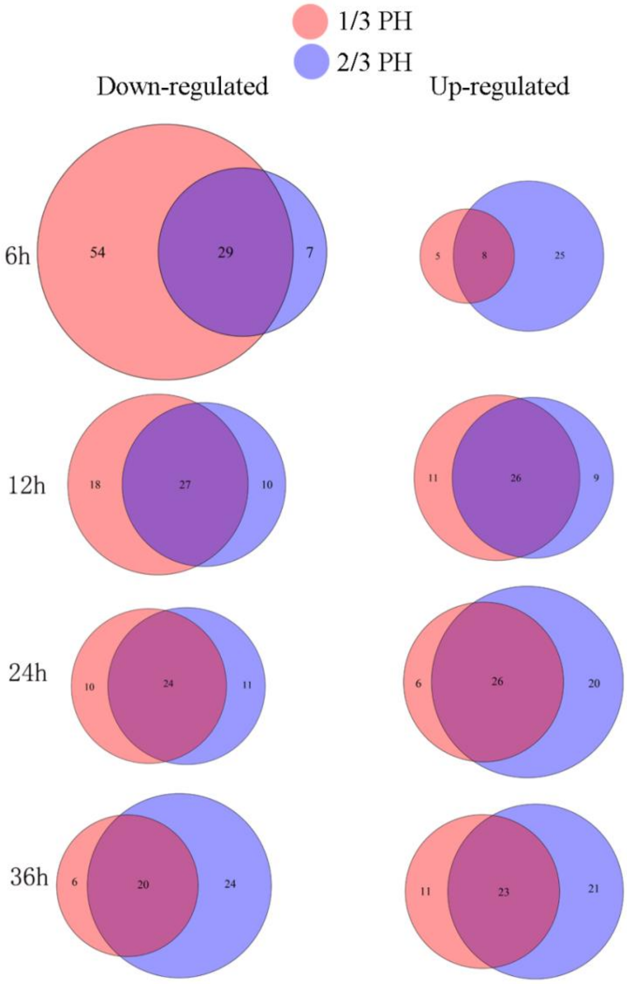

Supplementary Figure 2. Venn diagrams. Most of miRNAs whose expression is altered after 1/3 PH are similarly up- or down-regulated after 2/3 PH, except at 6 hours after PH.

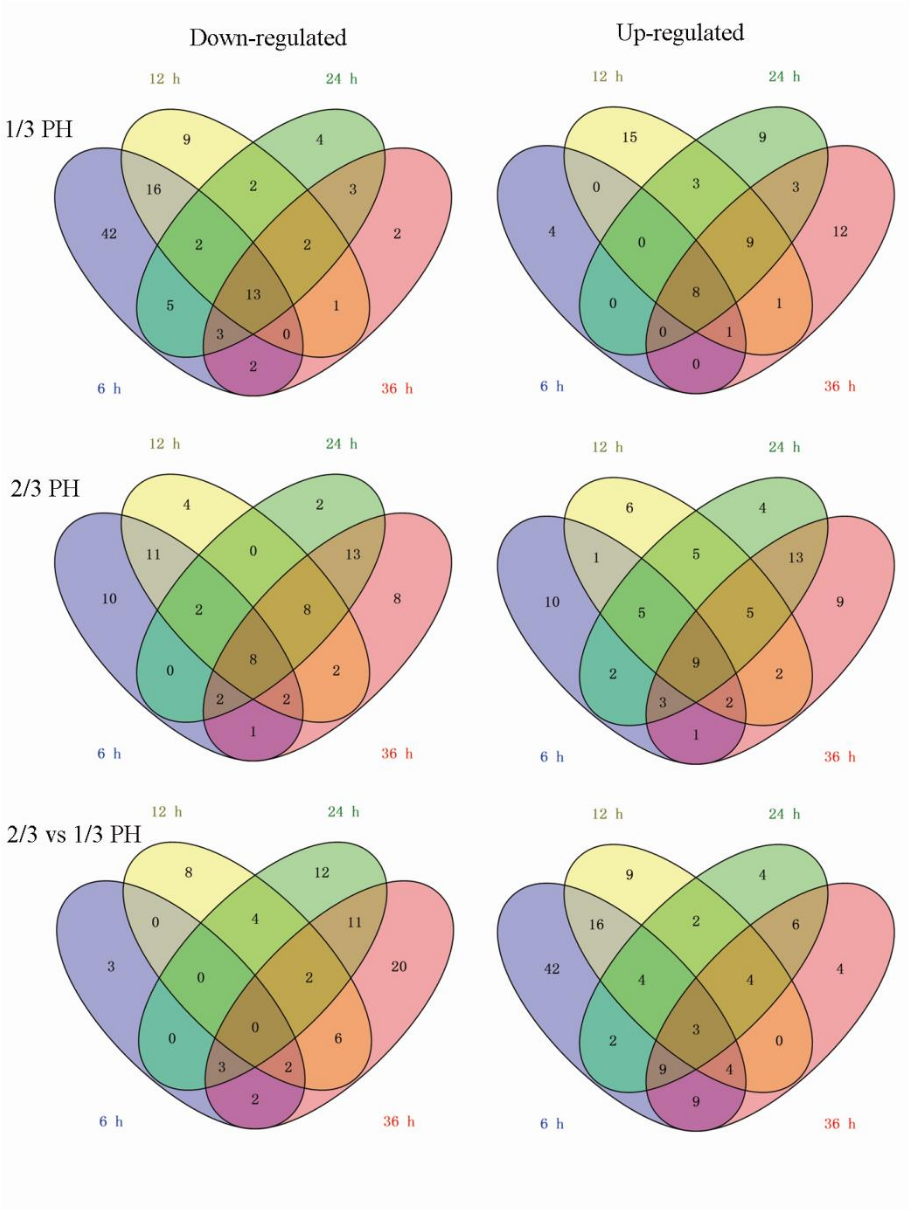

Supplementary Figure 3. Venn diagrams were draw according to their type of regulation (up/down), combined with the type of surgery. Most miRNAs showed a biphasic expression pattern.

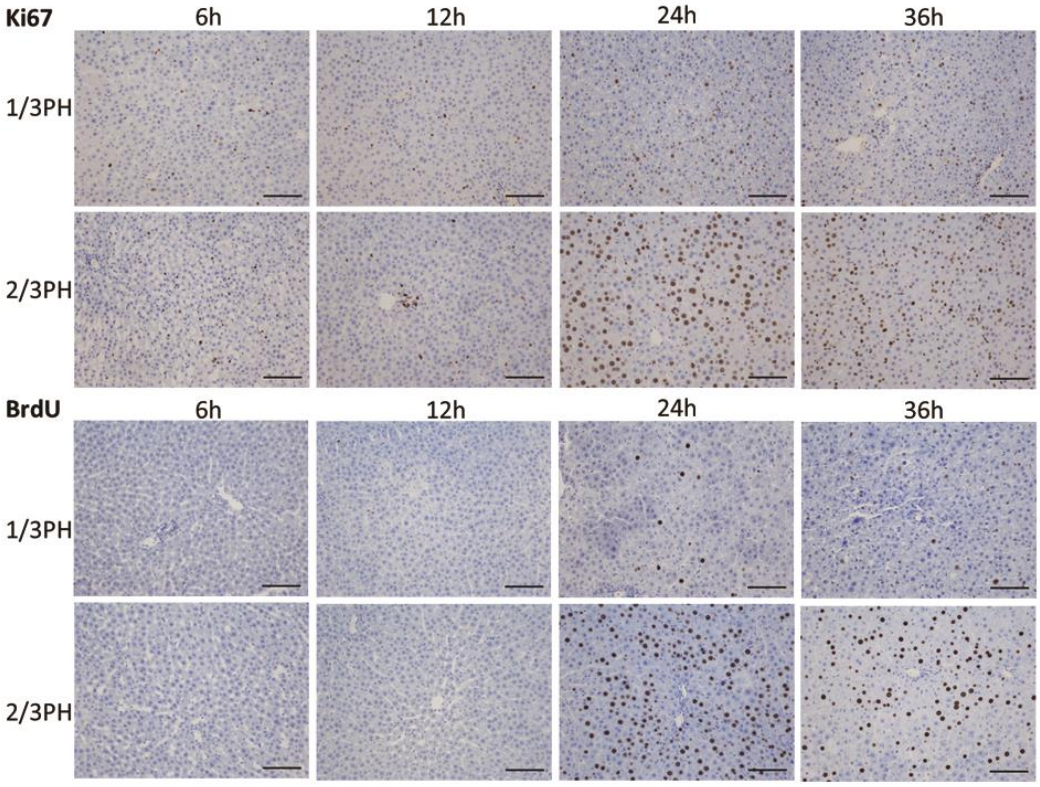

Supplementary Figure 4. Hepatocyte proliferation was assessed by mmunohistochemistry for Ki67 expression and BrdU incorporation. Staining for both markers showed that 1/3 PH caused a minimal replication; 2/3 induced a robust liver regeneration and a significant peak of DNA replication at 24 hours after PH. Scale bar = 100 μm; original magnification 200×, Olympus.

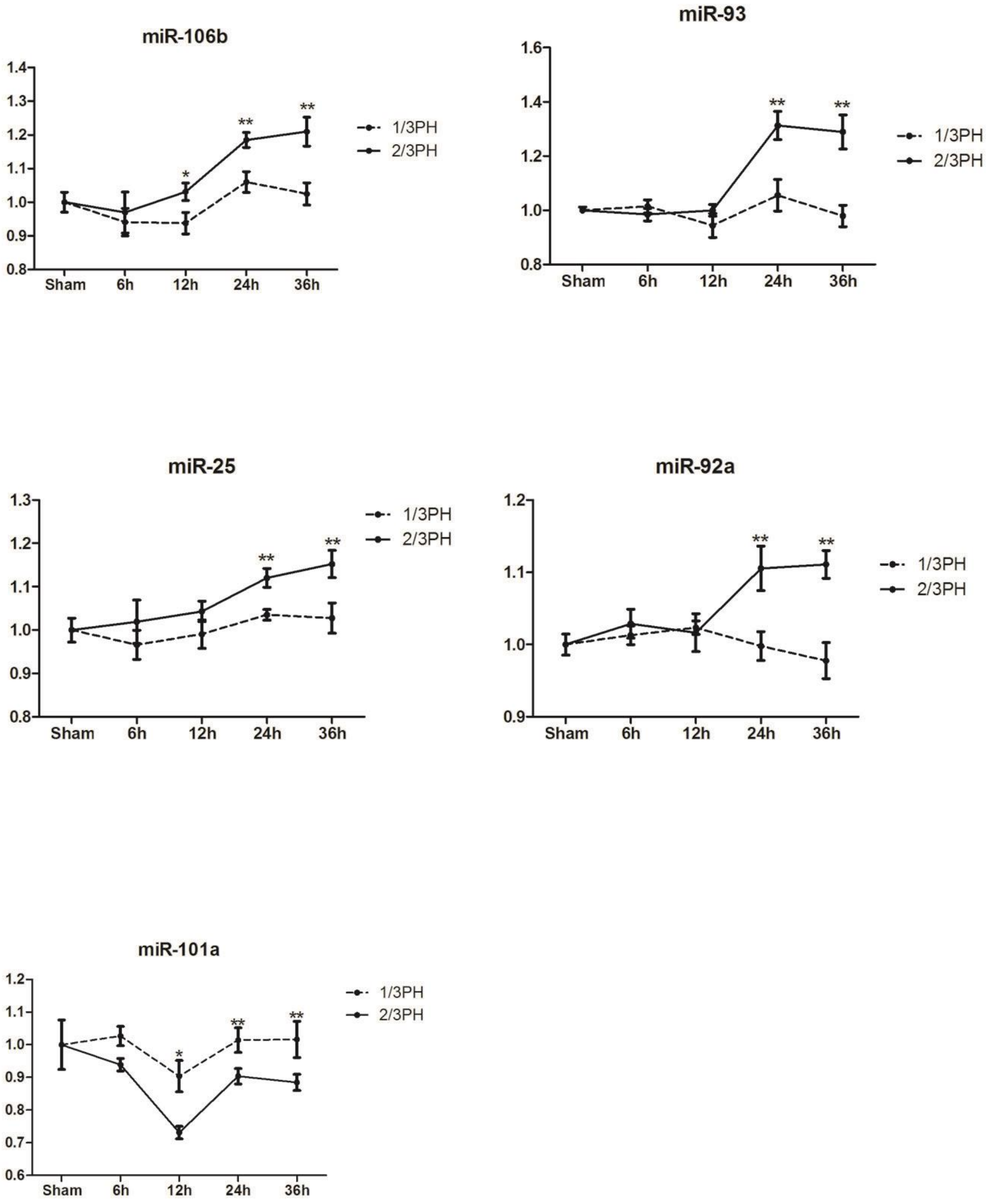

Supplementary Figure 5. The expression of miR-106b, miR -93, miR -25, miR -92a and miR -101a in normal liver and the remnant liver at serial times after 1/3 and 2/3 PH by real time-PCR. miR-106b, miR-93, miR-25 and miR-92a were found to be up-regulated at 24 and 36 hours after 2/3 PH, and miR-101a down-regulated at 12, 24 and 36 hours after 2/3 PH. The results represent findings obtained from 3 rats. Error bars represent the SD. *, $P < 0.05$ and **, $P < 0.005$.

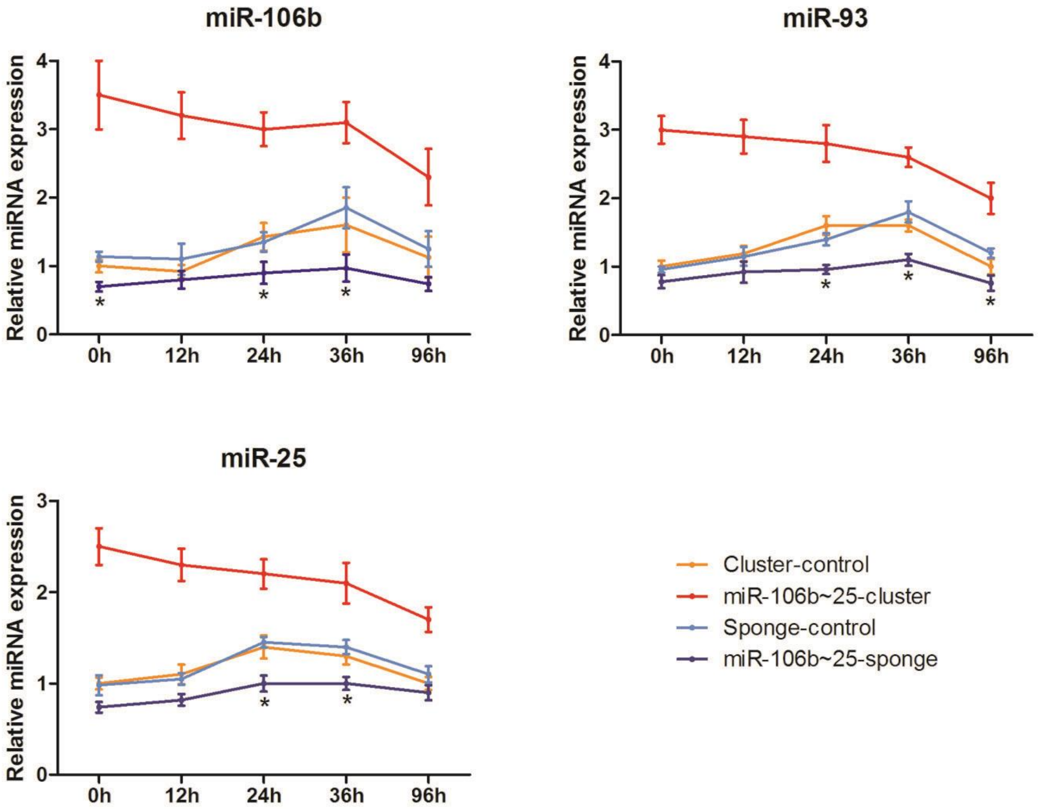

Supplementary Figure 6. The expression of miR-106b, miR-93 and miR-25 in the cluster- and sponge-treated rats compared with their respective controls after 2/3PH by real time-PCR. miR-106b, miR-93 and miR-25 were significantly overexpressed in cluster-treated rats than that in the controls and the sponge-treated rats at all times. miR-106b, miR-93 and miR-25 were significantly down-expressed in the sponge-treated rats than that in the controls at the timpoints marked with "*". The results represent findings obtained from 5 rats. Error bars represent the SD. *, P < 0.05.

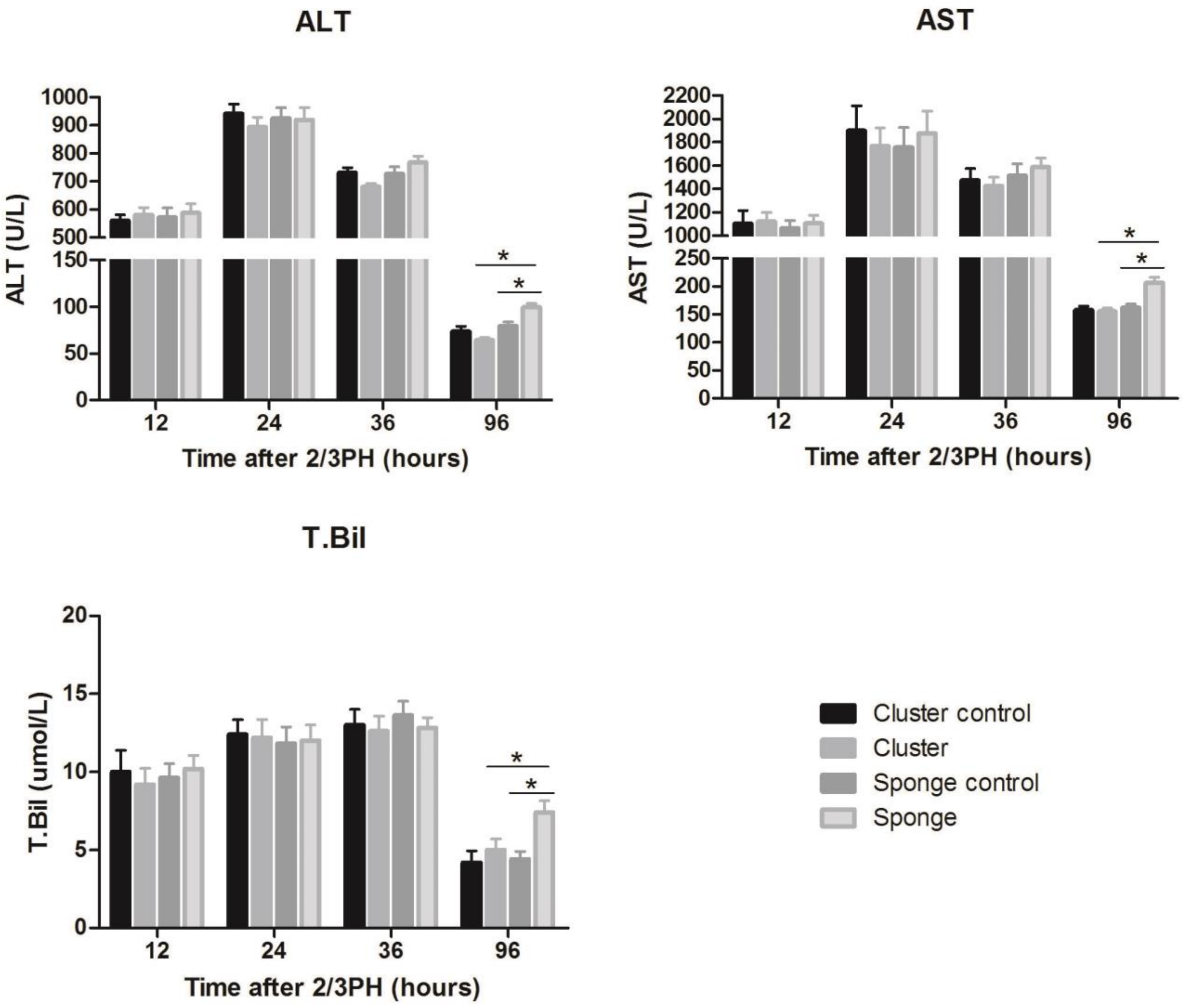

Supplementary Figure 7. The serum alanine aminotransferase (ALT), aspartate aminotransferase (AST), and the serum total bilirubin (T.Bil) levels affected by miR106b~25 cluster. ALT, AST, and T.Bil plasma concentration at 96 hours after 2/3 PH was significantly higher in the miR-sponge group compared to the control group and miR-cluster group. There were no significant differences observed among the four groups at other timeponts. The results represent findings obtained from 5 rats. All values are mean ± SEM. *, $P < 0.05$.

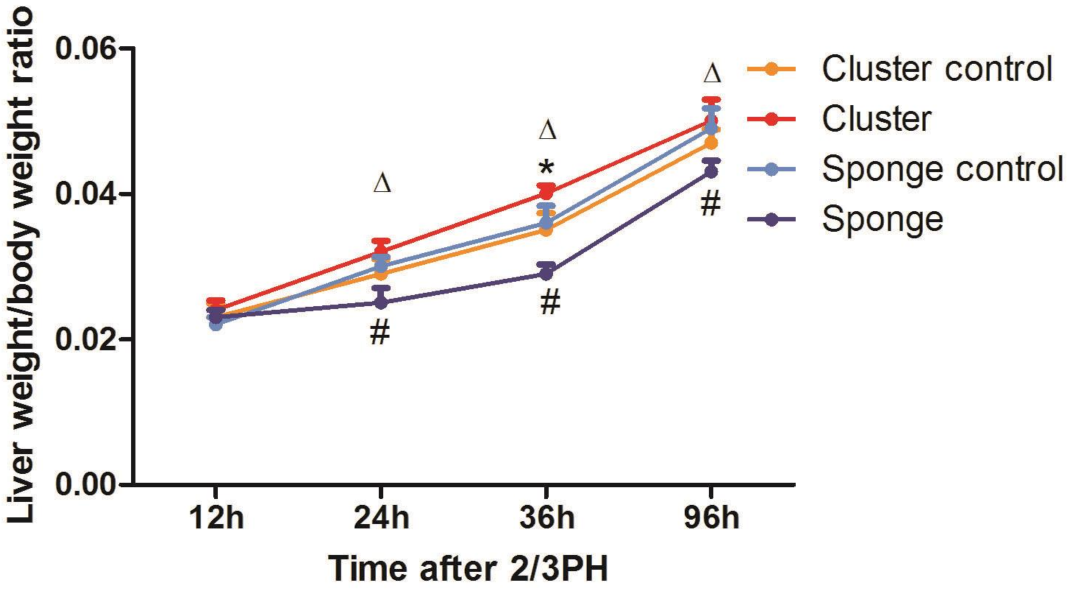

Supplementary Figure 8. The liver weight/body weight ratio in the miR-cluster- and miR-sponge-treated rats compared with their respective controls after 2/3PH. The ratio was significantly higher in miR-cluster overexpressing rats than the controls only at 36h after 2/3PH and remained significant lower in the miR-sponge-treated rats than the controls and the miR-cluster-treated rats from 24h to 96h after 2/3PH. *, P < 0.05 miR-cluster vs cluster control; #, P < 0.05 miR-sponge vs sponge control; △, P < 0.05 miR-sponge vs miR-cluster.

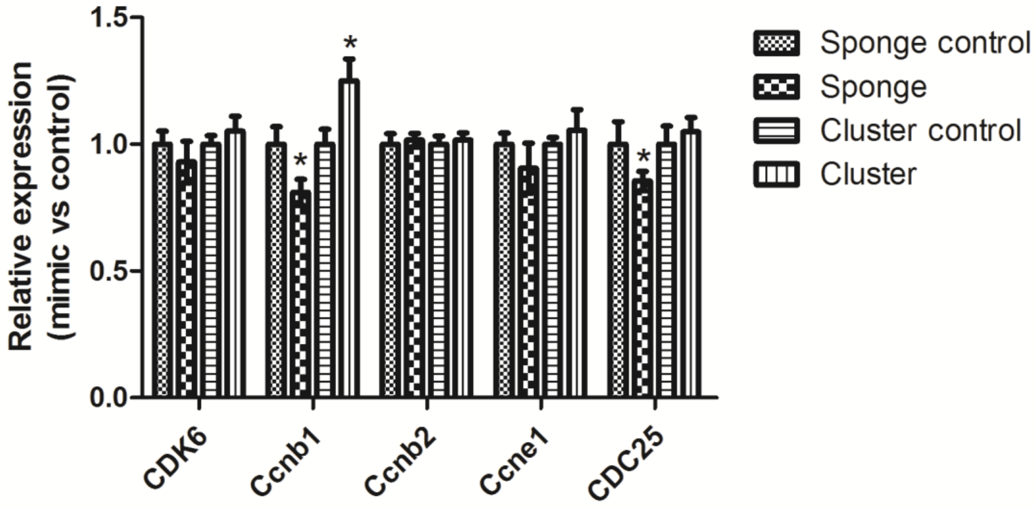

Supplementary Figure 9. The role of miR106b/25 on cyclins (Cdk6, Ccnb1, Ccnb2, Ccne1 and Cdc25) in the tissues of animals using real-time PCR at 24 hours after PH. The miR-106~25 cluster affect the expression of Ccnb1 and Cdc25.

Supplementary Tables

Supplementary Table 1. List of miRNAs detected in liver tissues using microarrays. All the data were normalized and FDR=20%. Normal livers: sm79normal_NS, sm65normal_NS, sm75normal_NS; 6 hours after 1/3 PH: s01m62_NS, s01m64_NS; 6 hours after 2/3 PH: s02m67_NS, s02m70B_NS, s02m76_NS; 12 hours after 1/3 PH: s03m2_NS, s03m5m_NS, s03m6_NS; 12 hours after 2/3 PH: s04m3_NS, s04m7_NS, s04m12B_NS; 24 hours after 1/3 PH: s05m20_NS, s05m23_NS, s05m24_NS; 24 hours after 2/3 PH: s06m26_NS, s06m29B_NS, s06m31B_NS; 36 hours after 1/3 PH: s07m45B_NS, s07m46_NS, s07m47_NS; 36 hours after 2/3 PH: s08m37B_NS, s08m39B_NS, s08m40_NS.

systematic_name	sm79normal_NS	sm65normal_NS	sm75normal_NS	s01m62_NS	s01m64_NS	s02m67_NS	s02m70B_NS	s02m76_NS	s03m2_NS	s03m5m_NS	s03m6_NS	s04m3_NS	s04m7_NS	s04m12B_NS	s05m20_NS	s05m23_NS	s05m24_NS	s06m26_NS	s06m29B_NS	s06m31B_NS	s07m45B_NS	s07m46_NS	s07m47_NS	s08m37B_NS	s08m39B_NS	s08m40_NS
rno-let-7a	10.51	10.51	10.51	10.51	10.51	10.51	10.51	10.51	10.72	10.72	10.72	10.72	10.72	10.72	10.51	10.51	10.51	10.72	10.51	10.72	10.51	10.51	10.51	10.72	10.72	10.72
rno-let-7b	10.04	9.79	10.04	9.91	10.04	10.04	10.04	9.79	9.91	9.91	9.91	9.91	9.91	9.91	9.66	9.79	9.79	9.91	9.79	9.79	9.91	10.04	9.79	9.91	9.79	9.66
rno-let-7b*	0.49	-3.32	3.30	-3.22	2.96	4.25	-3.14	-3.29	4.04	-2.85	3.35	3.56	3.63	-3.30	2.38	4.01	-3.31	0.27	3.27	4.25	4.08	-3.26	3.38	-2.98	3.35	-3.27
rno-let-7c	9.91	9.91	9.79	10.04	9.91	9.91	9.91	10.04	10.04	10.04	10.04	10.04	10.04	10.04	9.79	10.04	9.91	9.79	9.91	9.91	10.04	9.79	9.91	9.66	9.91	9.79
rno-let-7d	8.82	8.99	9.14	8.82	8.82	9.14	8.82	8.99	8.82	8.99	8.82	8.82	8.82	8.82	8.82	8.99	8.67	8.67	8.67	8.82	8.82	8.82	8.82	8.82	8.82	8.67
rno-let-7e	6.40	6.19	6.15	6.33	5.89	6.40	6.62	6.49	6.73	7.06	6.49	6.68	6.93	6.55	6.25	6.49	6.49	6.49	6.40	6.25	6.55	6.15	6.15	5.93	5.98	6.09
rno-let-7f	10.2	10.2	10.2	10.2	10.2	10.2	10.2	10.2	10.2	10.2	10.2	10.2	10.5	10.2	10.2	10.2	10.2	10.0	10.2	10.2	10.2	10.2	10.2	10.2	10.2	10.2

	6	6	6	6	6	6	6	6	6	6	6	6	1	6	6	6	6	4	6	6	6	6	6	6	6	6
rno-let-7i	7.80	7.39	7.67	7.80	7.51	7.80	7.88	7.61	7.88	7.74	7.88	7.80	8.16	7.56	7.74	7.74	7.88	7.96	7.80	7.61	7.74	7.67	7.61	7.96	7.88	7.56
rno-miR-1*	1.96	-3.32	0.49	2.24	-3.21	3.59	-3.14	-3.29	2.38	-2.85	3.27	4.12	3.05	-3.30	3.53	3.01	-3.31	2.44	3.56	3.16	3.53	1.15	3.33	2.57	2.96	-3.27
rno-miR-100	2.61	2.61	1.15	2.90	-3.21	2.48	-3.14	3.12	3.45	-2.85	2.44	3.19	3.16	2.61	2.24	3.48	3.42	3.01	2.38	2.84	2.38	3.12	0.72	-0.03	0.72	2.38
rno-miR-101a	4.57	5.39	5.16	4.61	6.68	4.39	3.12	4.97	4.57	-2.85	4.30	3.16	3.38	4.35	5.01	5.16	5.01	4.65	4.74	4.61	5.11	4.74	5.28	4.25	4.44	4.48
rno-miR-101b	7.16	7.80	7.80	7.44	6.62	6.99	6.86	7.28	7.16	7.16	7.34	7.11	6.68	7.28	7.96	7.80	7.56	7.67	7.67	7.67	7.80	7.61	7.80	7.67	7.80	7.80
rno-miR-103	6.19	6.15	5.98	6.15	5.78	6.25	6.25	6.25	6.33	5.49	6.25	6.25	6.33	6.40	6.15	6.25	6.19	6.25	6.15	6.33	6.49	6.09	6.33	5.98	6.15	6.25
rno-miR-106b	5.28	5.34	5.49	5.16	4.35	4.87	4.53	5.16	4.97	4.71	5.01	4.93	5.22	5.22	5.67	5.54	5.39	5.73	5.98	5.98	5.49	4.97	5.58	6.04	6.09	5.73
rno-miR-107	6.62	6.68	6.55	6.40	5.83	6.68	6.55	6.62	6.62	6.15	6.55	6.73	6.73	6.73	6.68	6.73	6.62	6.73	6.62	6.68	6.62	6.73	6.62	6.68	6.79	6.62
rno-miR-10a-5p	6.79	6.93	6.79	6.79	6.19	6.86	7.16	6.99	6.86	6.79	6.73	6.79	7.06	6.62	6.62	6.68	6.68	6.62	6.55	6.55	6.68	6.62	6.86	6.19	6.19	6.33
rno-miR-122	14.67	14.67	14.67	14.67	14.67	14.67	14.67	14.67	14.67	13.39	14.67	14.67	14.67	14.67	14.67	14.67	14.67	14.67	14.67	14.67	14.67	14.67	14.67	14.67	14.67	14.67
rno-miR-122*	7.11	7.67	7.44	7.16	6.79	7.34	7.44	7.80	7.34	8.47	7.51	7.39	7.39	7.74	7.34	7.34	7.34	6.86	7.28	7.34	7.39	7.16	7.34	7.16	7.24	7.74
rno-miR-1224	7.34	7.06	7.16	7.39	7.24	7.24	7.56	7.24	7.39	7.96	7.61	7.96	7.96	7.61	7.24	6.99	6.99	7.11	7.34	7.74	6.99	7.74	7.44	7.51	7.51	7.39
rno-miR-1249	3.53	-3.32	3.72	2.61	3.05	3.68	3.23	-3.29	4.08	-2.85	3.45	4.30	5.34	-3.30	2.61	3.63	-3.31	3.53	3.93	4.04	3.56	4.04	4.04	3.38	3.66	-3.27

rno-miR-125a-3p	3.38	-0.33	3.48	4.35	-3.21	-3.26	4.25	2.96	1.66	-2.85	4.65	2.57	4.12	4.77	2.48	-0.23	3.35	4.77	-3.24	1.15	1.66	5.06	3.42	5.06	3.72	4.81
rno-miR-125a-5p	4.21	3.53	3.66	4.12	3.66	4.12	4.08	4.01	4.35	4.39	3.97	3.63	3.93	3.97	3.93	3.97	4.21	3.66	3.72	4.08	4.12	3.63	3.68	3.33	3.33	3.38
rno-miR-125b-5p	6.93	7.24	6.93	6.93	6.25	7.06	7.28	7.39	7.67	7.11	7.16	7.34	7.28	7.67	7.06	7.24	7.39	6.99	6.86	7.16	7.16	6.99	6.79	6.33	6.73	7.11
rno-miR-126	8.67	8.67	8.57	8.57	8.67	8.57	8.57	8.57	8.47	8.16	8.67	8.47	8.57	8.47	8.57	8.67	8.57	8.57	8.57	8.47	8.57	8.67	8.57	8.29	8.47	8.47
rno-miR-128	2.24	1.41	-0.33	2.84	-3.21	-3.26	-3.14	1.41	-3.23	-2.85	-2.33	-3.29	-3.29	1.15	-3.25	-3.29	1.66	-0.33	-3.24	-3.25	-3.29	-3.26	2.31	-1.71	-0.23	-3.27
rno-miR-130a	6.55	6.55	6.49	6.73	5.98	6.62	6.73	6.93	6.99	6.93	6.86	6.86	6.86	6.99	6.73	6.62	6.79	6.79	6.79	6.62	6.73	6.33	6.55	7.34	6.93	7.06
rno-miR-139-3p	5.01	3.30	2.84	5.89	4.25	2.96	6.15	6.09	3.27	4.44	5.98	4.97	3.23	6.49	4.77	1.41	5.78	5.78	4.61	2.54	2.31	6.19	4.74	6.86	5.22	5.83
rno-miR-140	5.11	5.11	5.11	4.77	4.44	4.65	4.87	4.87	5.22	5.45	4.53	4.61	4.53	5.01	4.93	5.06	5.22	4.71	5.11	4.97	5.22	4.81	5.11	4.21	4.48	4.61
rno-miR-140*	3.45	4.35	4.04	3.45	3.33	3.48	3.53	3.66	4.25	4.17	2.94	2.96	3.56	3.68	3.23	3.68	3.97	3.42	3.09	3.45	3.72	3.38	3.19	2.38	2.90	3.45
rno-miR-142-3p	5.06	5.73	5.34	5.01	4.30	4.71	3.66	4.74	4.53	4.77	3.72	3.97	4.57	5.16	4.71	4.74	5.28	3.63	4.77	3.27	5.16	4.39	5.01	4.12	4.35	4.35
rno-miR-143	7.24	7.16	7.06	6.86	6.49	7.16	6.99	7.16	7.24	7.24	6.99	7.24	7.24	7.24	7.11	7.16	7.61	7.39	7.16	6.93	6.93	7.39	7.11	6.73	7.16	6.73
rno-miR-146a	5.73	5.93	5.67	5.54	5.11	6.09	6.09	6.04	5.89	5.98	5.58	5.89	6.25	5.89	5.45	5.58	5.67	5.28	5.67	5.22	6.33	5.28	5.83	4.71	5.73	5.54
rno-miR-148b-3p	3.09	4.39	4.01	2.96	3.09	2.94	3.27	4.21	2.84	-2.85	-0.73	-3.29	2.24	2.90	2.90	3.12	4.25	2.48	2.84	2.38	2.54	3.09	2.90	0.49	2.61	3.53

rno-miR-150	4.12	4.25	4.17	3.93	3.30	4.08	3.38	3.93	3.93	-2.85	3.05	3.38	4.17	4.17	3.45	3.59	4.01	3.33	3.38	3.23	3.59	3.35	3.35	2.61	3.19	3.30
rno-miR-150*	4.93	4.93	5.58	4.97	3.97	4.97	5.06	4.35	5.01	-2.85	5.67	4.87	5.49	5.06	5.28	5.22	4.65	5.34	4.65	5.45	4.97	5.67	4.93	5.73	5.34	5.11
rno-miR-151	6.49	6.49	6.40	6.49	5.93	6.55	6.79	6.79	6.04	6.68	5.78	6.40	6.09	5.93	5.89	6.19	6.15	6.15	5.89	6.04	5.78	6.55	6.49	5.54	5.89	5.93
rno-miR-152	3.12	3.68	3.45	3.23	2.48	3.05	3.09	3.42	3.97	-2.85	3.01	3.30	3.12	3.42	3.72	3.35	4.04	3.16	3.48	3.48	3.63	3.27	3.53	2.54	3.59	4.01
rno-miR-15b	7.39	7.28	7.28	7.74	7.16	7.39	7.67	7.44	7.44	7.56	7.67	7.44	7.80	7.80	7.51	7.51	7.51	7.44	8.04	7.56	7.11	7.24	7.28	8.57	8.57	8.57
rno-miR-16	8.99	8.47	8.99	8.99	8.99	8.82	8.99	8.47	9.14	8.67	8.99	8.99	9.14	9.14	9.14	9.14	9.14	8.99	8.99	9.14	9.14	8.99	9.14	9.14	9.46	8.99
rno-miR-17-5p	2.54	3.23	2.48	3.09	2.54	3.16	-3.14	3.05	3.12	-2.85	2.90	3.48	0.27	2.84	3.33	2.96	3.05	4.35	3.97	4.21	3.05	2.48	2.54	3.93	4.01	4.04
rno-miR-181a	3.59	4.21	2.96	3.63	3.35	3.63	3.72	4.39	3.66	-2.85	2.61	3.12	3.27	3.53	3.01	3.16	4.30	2.90	3.30	3.35	3.16	3.23	3.27	0.72	2.24	3.01
rno-miR-18a	-3.30	0.27	-0.73	-3.22	-3.21	-3.26	-3.14	-3.29	-3.23	-2.85	-2.16	-3.29	-3.29	1.66	3.12	1.15	2.31	3.97	4.08	3.97	2.84	-3.26	-3.29	4.17	4.12	3.66
rno-miR-191*	-1.13	-3.32	1.66	2.31	2.94	3.45	2.96	-3.29	2.31	-2.85	1.15	3.33	2.54	-3.30	3.35	2.94	-3.31	-2.13	3.23	2.48	3.30	-3.26	-0.03	-0.73	-1.71	-3.27
rno-miR-192	9.66	10.04	9.91	9.79	9.79	9.66	9.79	9.91	9.66	9.79	9.46	9.46	9.79	9.79	9.91	9.91	10.04	9.46	9.66	9.66	9.79	9.91	10.04	9.79	9.66	9.91
rno-miR-193	6.25	6.79	6.86	6.04	5.49	5.83	5.73	6.33	6.40	7.67	6.33	5.93	5.78	6.93	6.93	6.79	6.55	6.40	6.73	6.79	6.79	6.25	6.68	6.49	6.40	6.68
rno-miR-194	7.51	7.61	7.61	7.56	7.28	7.44	7.51	7.34	7.80	7.61	7.74	7.74	7.51	7.88	7.80	7.88	7.44	7.28	7.56	7.80	7.88	7.51	7.74	7.44	7.61	7.67
rno-miR-19	3.63	4.01	3.01	3.27	3.12	3.53	-3.1	3.33	3.53	4.01	3.42	3.59	3.30	4.01	4.01	4.17	4.44	5.22	4.71	4.35	3.27	3.33	3.63	4.77	4.30	4.21

49							4																			
rno-miR-195	8.16	7.44	8.04	8.04	7.88	7.88	8.16	7.88	7.74	7.28	7.80	7.56	7.74	7.51	7.61	7.67	7.24	7.61	7.51	7.51	7.67	7.96	7.56	7.24	7.28	7.24
rno-miR-199a-3p	5.45	5.54	5.28	5.45	4.61	5.67	5.39	5.67	5.93	5.28	4.77	5.45	5.89	5.28	5.39	5.49	6.33	6.33	5.58	5.78	5.67	5.49	5.54	5.22	5.11	5.01
rno-miR-199a-5p	3.05	3.12	0.27	3.16	-3.21	2.61	-3.14	3.19	4.01	-2.85	0.27	2.90	2.48	2.38	2.94	3.38	4.48	3.19	3.12	3.33	3.09	2.54	2.38	2.31	1.41	3.12
rno-miR-19a	2.57	4.97	4.74	3.35	2.31	2.57	-3.14	4.12	2.94	-2.85	2.84	3.01	-0.03	3.23	3.56	3.53	3.66	4.39	4.44	3.53	4.25	3.45	3.56	4.65	4.71	4.77
rno-miR-19b	8.29	8.82	8.67	8.47	7.74	7.96	7.80	8.04	7.96	7.51	8.04	8.04	7.61	8.04	8.67	8.16	8.29	8.82	8.82	8.67	8.47	8.47	8.47	8.67	8.67	8.82
rno-miR-200a	2.38	2.84	-1.52	-3.22	-3.21	-3.26	-3.14	2.90	3.23	-2.85	2.38	2.38	1.15	2.94	3.19	3.19	3.59	3.35	2.57	3.30	3.38	2.90	1.96	3.27	3.48	3.16
rno-miR-200b	4.53	4.71	3.38	4.25	3.59	4.21	4.57	4.93	5.49	6.04	5.34	5.11	4.48	4.71	5.06	5.34	5.16	5.16	4.87	5.34	5.34	4.35	4.01	5.83	5.78	5.06
rno-miR-202*	5.83	5.45	5.89	6.25	5.16	5.58	6.33	6.15	5.58	4.21	6.62	6.33	5.45	6.15	6.49	5.78	5.98	6.68	5.93	5.89	5.54	6.86	6.09	7.11	6.62	6.55
rno-miR-20a	7.06	6.86	6.99	7.28	6.40	7.11	7.06	6.68	7.06	6.73	7.44	7.61	6.62	6.79	7.39	7.11	6.73	8.04	7.88	7.96	6.86	7.11	6.93	8.04	7.67	7.51
rno-miR-20b-5p	4.87	5.16	5.06	4.93	4.48	5.39	4.30	4.81	5.16	4.65	5.06	5.54	4.21	4.97	5.22	5.11	4.97	5.83	5.83	5.93	5.01	4.61	4.81	5.28	5.45	5.34
rno-miR-21	11.43	11.74	11.74	12.15	11.43	11.74	11.74	11.74	11.21	11.43	11.74	11.74	11.02	11.74	12.15	11.74	11.74	12.42	12.15	12.42	12.15	12.15	12.15	12.42	12.15	11.74
rno-miR-210	3.56	4.12	3.33	3.38	3.38	2.84	3.42	4.08	3.16	-2.85	2.57	2.84	2.90	3.05	3.48	3.27	3.38	3.12	3.19	3.72	2.61	2.96	3.01	3.30	2.48	2.90
rno-miR-21	5.93	5.06	6.09	6.09	5.22	5.54	6.19	5.89	5.45	3.93	6.93	6.19	5.28	5.73	5.83	5.67	5.58	6.04	5.49	4.93	5.45	6.68	6.19	6.62	6.33	6.40

2																										
rno-miR-214	4.01	3.97	3.05	3.68	3.23	3.97	3.68	4.30	4.77	4.30	3.16	3.45	4.25	4.08	3.42	4.65	5.06	4.48	3.53	4.77	3.66	3.59	3.05	3.97	3.16	3.72
rno-miR-22	10.72	11.02	11.02	11.02	10.72	10.72	10.72	10.72	10.51	11.02	10.51	10.51	10.26	10.51	10.72	10.72	11.02	10.51	10.72	10.51	10.72	11.21	10.72	10.51	10.51	10.51
rno-miR-221	-0.03	1.66	-3.32	-3.22	-3.21	1.15	-3.14	2.94	2.44	-2.85	-1.71	0.49	-3.29	-0.73	-3.25	0.27	2.48	0.49	1.96	2.24	-3.29	-3.26	-3.29	1.41	0.27	2.94
rno-miR-223	5.49	5.83	5.45	5.93	5.54	5.89	5.54	5.73	5.34	6.55	5.49	5.49	5.83	5.83	5.54	5.39	5.89	4.74	5.45	5.73	5.83	5.01	5.39	5.58	5.39	5.28
rno-miR-23a	7.28	7.56	7.24	7.11	7.11	7.51	7.34	7.67	7.51	7.88	7.06	7.16	7.34	7.44	6.86	7.06	7.74	7.16	6.99	7.06	7.28	7.06	7.24	6.79	7.06	7.16
rno-miR-23b	8.47	8.57	8.29	8.29	8.29	8.47	8.47	8.82	8.57	9.14	8.47	8.67	8.47	8.67	8.29	8.29	8.82	8.29	8.29	8.29	8.29	8.29	8.29	8.16	8.16	8.29
rno-miR-24	7.74	7.51	7.39	7.51	7.39	7.74	7.61	7.74	7.61	7.39	7.39	7.51	7.67	7.39	7.44	7.56	7.67	7.51	7.39	7.44	7.51	7.44	7.51	7.39	7.44	7.44
rno-miR-25	6.09	6.04	5.78	5.98	5.45	5.98	6.04	5.98	6.19	5.54	6.19	6.04	6.40	6.09	6.04	5.89	5.93	6.55	6.68	6.49	6.09	5.73	5.73	6.40	6.55	6.49
rno-miR-26a	8.57	9.14	8.82	8.67	8.57	8.67	8.67	8.67	8.67	8.82	8.57	8.57	8.67	8.57	8.47	8.57	8.47	8.16	8.16	8.16	8.67	8.57	8.67	7.80	8.04	8.04
rno-miR-26b	7.88	8.16	7.88	7.88	7.67	8.04	8.04	8.16	8.04	8.04	7.96	7.88	8.04	7.96	7.88	7.96	7.96	7.56	7.74	7.88	8.04	7.88	8.16	7.56	7.74	7.88
rno-miR-27a	6.86	6.62	6.73	6.62	6.04	6.49	6.40	6.55	6.79	7.34	6.40	6.62	6.55	6.68	6.79	6.86	6.93	7.06	7.06	7.11	7.24	6.93	6.99	6.93	6.99	6.93
rno-miR-27b	7.56	7.34	7.34	7.24	6.99	7.56	7.39	7.56	7.56	7.44	7.56	7.67	7.56	7.34	7.56	7.44	7.28	7.34	7.61	7.39	7.44	7.34	7.39	7.06	7.39	7.28
rno-miR-28	4.74	5.01	5.01	4.71	3.72	5.01	4.35	5.01	4.44	4.81	4.17	4.04	4.39	4.30	4.30	4.93	4.77	4.44	5.01	4.44	4.81	4.71	4.97	4.53	4.93	4.65
rno-miR-290	4.81	4.61	4.97	4.81	4.01	4.53	4.71	4.25	4.61	-2.85	4.97	5.06	4.77	4.65	4.87	5.01	4.35	4.81	4.53	4.71	4.74	5.39	4.87	4.93	5.06	4.74

rno-miR-296*	2.48	-3.32	0.72	2.48	-3.21	3.56	-3.14	-3.29	2.90	-2.85	3.30	3.93	4.61	-3.30	2.44	3.33	-3.31	3.23	2.94	3.05	3.42	3.93	3.93	3.05	3.23	-3.27
rno-miR-298	-3.30	-3.32	-1.71	-3.22	2.61	3.23	-3.14	-3.29	1.96	-2.85	0.72	-0.23	3.09	-3.30	1.15	3.09	-3.31	-0.23	1.66	1.66	2.90	-3.26	-0.33	-3.32	-1.92	-3.27
rno-miR-2985	4.44	3.33	4.39	4.57	4.65	5.22	4.44	3.45	4.74	5.89	4.44	5.28	5.06	3.27	5.16	4.87	3.16	4.21	4.97	5.16	5.06	4.65	4.77	4.01	4.65	3.33
rno-miR-29a	9.46	9.66	9.66	9.66	9.46	9.46	9.14	9.46	9.46	8.57	9.14	9.14	8.99	9.46	9.46	9.46	9.46	9.66	9.46	9.46	9.66	9.46	9.66	9.46	9.14	9.14
rno-miR-29b	6.99	8.04	7.56	7.06	6.15	6.79	6.49	6.73	6.68	6.49	6.68	6.49	6.49	7.06	7.28	7.39	7.11	7.80	7.44	7.24	7.61	7.56	7.67	7.88	7.56	7.61
rno-miR-29c	7.61	7.88	7.74	7.67	6.73	7.28	6.93	7.11	7.11	6.09	7.11	6.99	6.79	7.11	7.67	7.61	7.16	7.74	7.24	7.28	7.56	8.04	8.04	7.61	7.34	7.34
rno-miR-3085	3.72	3.45	4.12	4.04	4.08	4.74	4.21	3.27	4.39	5.83	4.08	4.44	4.87	3.72	4.44	4.53	3.30	3.68	4.35	4.65	4.65	4.30	4.17	3.53	4.04	3.56
rno-miR-30a	6.68	6.99	7.11	6.68	6.09	6.73	6.68	6.86	6.93	6.62	6.79	6.93	6.99	6.86	6.99	6.93	6.86	6.93	6.93	6.86	7.06	6.79	7.06	6.55	6.86	6.86
rno-miR-30a*	4.65	4.77	4.48	4.48	3.56	4.77	4.93	5.11	4.93	5.73	4.39	4.53	4.44	4.81	4.53	4.61	4.81	4.04	4.57	4.53	4.57	4.25	4.65	3.59	3.93	4.25
rno-miR-30b-3p	-1.52	-1.52	-1.92	-3.22	-3.21	0.72	2.84	2.38	2.24	-2.85	-1.13	-0.33	2.31	2.48	-3.25	-0.33	1.41	-1.71	-3.24	-3.25	-0.23	-3.26	-0.23	-2.98	-3.31	2.44
rno-miR-30b-5p	6.33	6.40	6.33	5.83	5.73	6.33	5.67	6.40	6.55	6.19	5.83	6.15	6.19	5.98	6.40	6.33	6.40	5.54	6.09	6.40	6.40	5.58	6.25	5.45	6.25	5.89
rno-miR-30c	5.89	6.33	6.04	5.78	5.34	6.04	5.93	6.19	6.25	6.86	6.04	5.98	6.04	6.19	6.09	6.04	6.25	5.93	6.25	5.83	6.19	5.89	6.04	5.89	6.04	6.15
rno-miR-30d	5.58	6.25	5.73	5.49	5.06	5.93	5.83	5.93	5.73	6.40	5.54	5.78	5.67	6.25	5.93	5.98	6.09	5.67	5.78	6.09	6.15	5.78	5.67	5.49	5.83	6.19

rno-miR-30e	5.54	5.98	5.83	5.58	4.57	5.34	4.97	5.45	5.39	5.01	5.22	5.22	4.93	5.67	5.73	5.73	5.73	5.49	5.73	5.39	5.73	5.54	5.78	5.34	5.49	5.45
rno-miR-30e*	3.23	3.66	3.19	3.30	3.27	3.30	3.59	3.68	3.56	4.74	2.48	2.54	2.96	3.35	3.09	2.84	3.33	2.31	3.05	2.90	2.48	3.05	3.09	2.24	1.15	3.42
rno-miR-31	5.39	5.49	5.54	5.11	4.71	5.73	5.22	5.58	5.67	4.61	5.39	5.73	5.54	5.58	5.78	5.45	5.49	5.58	5.54	5.67	5.58	5.16	5.49	5.16	5.58	5.67
rno-miR-31*	-3.30	2.31	-0.03	-3.22	-3.21	2.31	-3.14	2.57	2.96	-2.85	0.49	1.96	-3.29	2.31	-3.25	-3.29	1.15	0.72	2.31	-3.25	0.27	-3.26	-3.29	-1.52	-0.03	2.61
rno-miR-320	3.48	4.17	4.44	3.05	-3.21	2.38	3.05	3.35	3.01	-2.85	4.12	1.66	3.42	3.59	4.08	4.30	3.45	4.08	3.63	3.09	3.97	4.53	4.08	4.30	4.21	4.30
rno-miR-322	5.34	5.22	5.39	5.34	4.74	5.28	5.16	5.54	5.54	4.97	5.11	5.16	5.39	5.39	5.34	5.28	5.45	5.06	5.34	5.54	5.28	4.93	5.16	4.61	4.74	4.71
rno-miR-324-3p	5.98	5.78	6.62	5.73	5.28	6.15	5.89	5.22	6.09	5.11	6.15	6.09	6.15	5.34	6.33	6.55	5.34	5.89	6.04	6.19	6.04	5.98	5.98	6.25	6.49	6.04
rno-miR-328a	2.90	-1.71	-0.23	-3.22	-3.21	2.44	3.16	2.24	2.48	-2.85	-0.03	2.24	1.96	2.54	-3.25	0.72	-1.13	-0.03	-3.24	-3.25	0.49	-3.26	-3.29	-1.92	-3.31	1.41
rno-miR-331	1.66	0.72	-2.33	-3.22	-3.21	-3.26	-3.14	1.96	-3.23	-2.85	-3.32	-3.29	-3.29	-3.30	-3.25	-3.29	-1.52	-3.31	-3.24	-3.25	-3.29	2.31	-3.29	-3.32	-3.31	-3.27
rno-miR-335	2.44	4.04	3.09	3.33	-3.21	3.19	3.35	4.61	3.68	3.97	3.48	3.23	3.33	3.56	3.16	3.42	4.53	2.57	3.16	3.38	3.19	2.44	3.12	3.19	3.42	3.19
rno-miR-338	3.93	3.56	3.56	3.66	-3.21	1.66	-3.14	3.48	-3.23	-2.85	-2.32	-3.29	-3.29	-3.30	-3.25	-3.29	2.61	-3.31	-3.24	-3.25	-3.29	-3.26	3.59	-3.32	-3.31	-3.27
rno-miR-342-3p	3.30	3.48	2.94	3.12	3.01	3.01	3.33	3.09	3.35	-2.85	1.96	1.41	4.35	4.12	2.84	3.05	3.93	1.96	2.61	3.01	4.35	2.84	2.61	-1.13	2.38	2.57
rno-miR-345-3p	4.25	4.30	4.61	5.39	2.24	3.12	4.77	4.44	3.63	-2.85	4.93	4.01	2.94	4.93	4.74	4.44	4.57	5.11	4.12	4.17	4.39	5.34	4.71	5.11	4.81	5.22
rno-miR-34	4.17	4.65	4.57	4.21	3.53	4.35	3.97	4.53	4.30	4.35	3.93	4.57	3.35	4.21	4.25	4.08	4.71	4.53	4.93	4.81	4.17	4.08	4.35	4.81	4.77	4.87

5-5p																										
rno-miR-347	3.35	3.01	3.23	3.53	2.84	2.54	3.45	3.97	2.61	-2.85	3.68	3.09	0.72	4.04	2.54	2.57	3.23	2.84	-3.24	-3.25	2.44	3.72	3.23	3.66	2.84	4.08
rno-miR-34a	5.16	5.28	5.22	5.28	4.87	5.16	5.49	5.28	5.98	5.34	6.09	5.83	5.58	5.49	6.19	6.15	5.83	6.09	6.49	6.73	6.25	5.11	5.89	7.28	6.68	6.99
rno-miR-352	2.96	3.16	2.31	2.94	2.38	3.09	3.48	3.38	3.30	-2.85	2.54	2.48	2.44	3.19	-3.25	2.38	2.96	2.24	2.54	2.94	2.57	2.94	2.57	1.96	2.31	3.23
rno-miR-3544	-3.30	-3.32	2.57	-3.22	2.57	4.01	-3.14	-3.29	2.57	-2.85	3.12	4.21	3.48	-3.30	3.97	3.56	-3.31	3.05	3.42	4.01	4.04	2.24	1.41	2.48	3.12	-3.27
rno-miR-3549	-0.33	-3.32	3.53	3.59	3.63	4.30	-3.14	-3.29	3.72	-2.85	3.53	4.08	3.72	-3.30	4.39	4.04	-3.31	3.27	4.17	4.30	4.21	3.68	3.72	3.01	3.63	-3.27
rno-miR-3559-5p	4.30	4.87	4.81	4.44	6.55	4.17	4.04	4.48	4.71	-2.85	4.61	4.25	3.66	4.25	5.11	4.71	4.93	4.93	5.28	5.01	4.93	4.48	5.22	4.87	5.16	5.16
rno-miR-3561-5p	0.72	2.38	3.59	3.72	-3.21	-3.26	3.19	2.31	-3.23	-2.85	4.01	-3.29	-3.29	3.66	-3.25	1.66	0.49	4.25	-3.24	-3.25	-3.29	4.44	1.66	4.44	3.56	4.12
rno-miR-3562	4.71	3.93	4.65	5.06	3.93	4.04	5.11	4.04	4.21	-2.85	5.28	4.74	4.01	5.11	4.81	4.25	4.12	5.39	4.39	4.48	3.93	5.83	4.61	6.15	5.01	5.39
rno-miR-3573-3p	3.19	2.96	3.42	-3.22	-3.21	3.38	-3.14	-3.29	-3.23	4.87	3.33	4.17	4.04	3.16	2.31	3.30	2.84	3.30	3.59	3.68	3.33	3.56	3.48	3.23	3.27	1.96
rno-miR-3588	7.44	5.58	6.19	7.34	7.06	7.61	7.24	5.83	6.49	4.48	7.28	7.06	7.11	5.45	5.58	6.09	4.17	4.97	5.39	5.58	5.98	6.40	6.40	6.09	4.53	4.93
rno-miR-361	3.33	4.08	3.97	3.42	3.16	3.35	3.63	3.72	3.42	-2.85	2.96	3.35	3.01	3.30	3.38	3.66	3.53	3.38	3.45	3.56	3.35	3.53	3.97	3.42	3.38	3.59
rno-miR-362*	1.15	2.54	2.38	2.44	-3.21	-3.26	-3.14	0.49	-3.23	-2.85	-3.32	-3.29	-3.29	-3.30	1.96	2.31	2.38	1.15	2.48	2.31	2.24	2.57	2.48	0.27	1.96	2.31
rno-miR-36	4.97	5.67	4.87	4.87	4.53	5.11	5.01	5.78	5.06	5.67	4.87	5.34	4.71	5.78	4.97	4.97	5.54	4.61	5.16	5.11	4.87	4.77	5.06	4.48	4.87	5.58

5																										
rno-miR-378	6.73	6.73	6.68	6.55	6.33	6.93	7.11	7.06	5.78	6.25	5.45	6.55	5.73	6.04	5.98	5.83	6.04	6.19	6.19	6.15	5.39	6.49	6.73	5.39	5.93	5.98
rno-miR-378*	4.39	4.53	4.30	3.56	4.17	4.57	4.01	4.65	3.09	-2.85	-0.23	3.05	1.66	3.93	3.05	2.54	3.27	1.66	2.44	2.57	1.96	3.30	4.39	1.15	0.49	2.84
rno-miR-409-3p	3.66	3.09	4.08	4.17	4.12	4.48	-3.14	3.16	3.48	5.39	3.66	4.35	4.08	3.48	4.57	4.48	2.90	3.59	4.30	4.57	4.61	4.12	4.30	3.56	4.17	3.35
rno-miR-425	2.31	2.48	1.96	-3.22	-3.21	2.24	-3.14	2.44	-3.23	-2.85	-1.52	1.15	1.41	1.41	-3.25	1.96	2.44	2.54	2.24	2.44	0.72	-3.26	2.24	2.96	2.44	2.96
rno-miR-429	2.84	1.96	-3.32	2.54	-3.21	-3.26	-3.14	2.54	3.59	4.04	3.59	2.94	2.38	2.44	3.63	3.45	3.56	4.12	2.96	3.93	3.48	2.61	1.15	4.08	4.25	3.09
rno-miR-451	6.04	6.09	5.93	6.99	5.58	5.06	5.34	5.34	6.15	5.93	5.16	5.39	7.16	6.33	6.55	6.40	7.80	5.98	6.33	5.28	5.93	6.04	5.45	5.01	5.54	5.78
rno-miR-455*	0.27	-0.03	-2.53	-3.22	-3.21	-3.26	-3.14	2.84	-3.23	-2.85	-1.74	0.27	-3.29	-0.23	-3.25	-3.29	-1.74	-3.31	-3.24	-3.25	-3.29	-3.26	-3.29	-3.32	-3.31	-3.27
rno-miR-466b	5.67	2.44	3.16	3.97	5.01	5.49	4.17	3.23	4.48	6.33	5.93	4.77	5.11	4.74	2.57	2.44	-0.03	-3.31	-3.24	-3.25	2.94	-3.26	-3.29	3.09	-0.73	2.54
rno-miR-466b-1*	-3.30	3.35	3.12	-3.22	-3.21	0.49	2.90	-0.73	-3.23	4.25	-3.32	-3.29	-0.33	3.09	-3.25	-0.73	-1.92	-3.31	-3.24	-3.25	-0.33	-3.26	-3.29	-2.53	-1.74	0.72
rno-miR-466b-2*	-3.30	3.27	2.24	-3.22	-3.21	-3.26	-3.14	-3.29	-3.23	-2.85	-3.32	-3.29	-3.29	-3.30	-3.25	-3.29	0.27	-3.31	-3.24	-3.25	-0.03	-3.26	-3.29	2.90	2.54	3.63
rno-miR-494	4.61	4.81	4.77	4.30	4.39	4.61	4.39	4.77	4.87	5.16	4.25	5.01	5.01	4.48	4.61	4.81	4.87	4.17	5.06	5.49	4.77	4.87	5.34	4.57	5.28	4.53
rno-miR-497	7.67	7.11	7.51	7.61	7.34	7.67	7.74	7.51	7.28	6.99	7.24	7.28	7.44	7.16	7.16	7.28	7.06	7.24	7.11	6.99	7.34	7.28	7.16	6.99	7.11	6.79
rno-miR-50	6.15	4.48	4.93	6.19	5.39	6.19	5.98	5.39	5.28	-2.8	5.73	5.58	5.93	4.87	3.59	4.57	3.68	2.94	2.90	2.96	4.44	4.57	4.57	4.35	3.01	3.68

0										5																
rno-miR-511*	4.77	4.57	4.25	4.53	4.21	4.93	4.61	4.57	4.65	4.57	4.35	4.39	4.74	4.61	4.17	4.39	4.61	3.93	4.21	3.42	4.30	3.97	4.21	3.48	3.45	3.48
rno-miR-532-5p	3.16	2.90	2.90	3.19	2.44	2.90	-3.14	3.01	3.05	-2.85	1.41	2.44	2.61	1.96	3.27	3.23	3.01	3.09	3.01	3.19	3.23	3.19	3.16	2.94	2.94	3.05
rno-miR-547	-3.30	-0.23	2.54	-3.22	6.86	-3.26	-3.14	1.66	-3.23	-2.85	3.23	-3.29	-3.29	3.45	-3.25	-3.29	2.24	3.56	-3.24	-3.25	-3.29	4.21	-3.29	4.04	3.05	3.97
rno-miR-582*	-0.73	-3.32	3.27	2.38	3.48	3.93	2.94	-3.29	3.19	-2.85	3.38	3.66	3.45	-3.30	4.12	3.72	-3.31	2.61	3.66	4.12	4.01	-3.26	3.45	2.84	3.53	-3.27
rno-miR-652	5.22	4.44	4.71	5.22	4.77	5.45	5.28	5.06	5.11	4.53	4.81	4.71	5.16	4.44	3.68	4.12	4.39	4.01	4.04	3.63	4.48	3.66	4.12	3.45	3.68	3.93
rno-miR-652*	5.78	5.89	6.25	5.67	4.97	5.78	5.45	5.49	5.83	5.22	5.89	5.67	5.98	5.54	5.49	5.93	5.11	5.01	5.22	5.06	5.89	5.93	5.93	5.67	5.67	5.49
rno-miR-674-3p	-3.30	-2.60	-2.57	-3.22	-3.21	-3.26	-3.14	-0.33	-3.23	-2.85	-3.15	-3.29	-3.29	-3.30	-3.25	-3.29	-2.13	-2.33	1.41	-3.25	-0.73	-3.26	-3.29	-2.13	-1.13	1.15
rno-miR-702-3p	3.42	2.57	3.68	4.08	7.96	4.44	-3.14	-3.29	4.12	5.06	4.04	4.48	3.97	-0.33	4.48	4.35	-3.31	3.72	4.25	4.39	4.53	4.17	4.25	3.63	4.08	-3.27
rno-miR-743a	3.97	2.94	4.21	4.74	-3.21	-3.26	4.81	4.17	-3.23	-2.85	4.74	3.72	-3.29	4.39	4.35	2.61	4.08	5.45	4.01	-3.25	1.41	5.45	4.53	5.78	4.57	4.97
rno-miR-7a	3.01	3.38	3.35	3.48	3.19	3.42	3.93	3.30	3.38	4.12	2.31	3.27	2.57	2.96	3.30	2.48	3.19	3.45	3.35	3.12	2.96	3.16	2.94	3.72	3.97	4.17
rno-miR-872	2.94	3.63	1.41	3.01	2.90	3.27	3.30	3.56	3.33	-2.85	2.24	2.61	2.84	2.57	2.96	2.90	2.94	3.48	3.33	2.61	3.01	3.01	2.84	3.35	2.57	3.27
rno-miR-878	4.04	3.59	4.35	4.65	-3.21	-3.26	4.65	3.63	-3.23	-2.85	4.71	3.53	-3.29	4.53	4.21	4.21	3.48	4.57	3.68	3.59	3.12	5.22	4.48	4.97	4.61	4.57
rno-miR-92a	7.96	7.96	8.16	8.16	8.16	8.29	8.29	8.29	8.16	8.29	8.29	8.29	7.88	8.16	8.04	8.04	8.04	8.47	8.47	8.57	7.96	7.80	7.88	8.47	8.29	8.16

rno-miR-93	3.68	3.72	3.63	4.01	3.42	3.72	3.56	3.53	4.17	-2.85	3.56	3.68	3.53	3.63	4.04	3.93	3.63	4.87	4.81	4.74	3.68	3.42	3.66	4.74	4.97	4.44
rno-miR-96	3.27	3.19	-1.74	-3.22	-3.21	-3.26	-3.14	2.61	2.54	-2.85	1.66	-3.29	-3.29	-1.13	-3.25	2.24	3.12	2.38	-3.24	-3.25	-3.29	1.96	2.44	-3.32	3.30	2.24
rno-miR-98	4.35	4.74	4.53	4.39	4.04	4.81	4.74	4.71	4.81	4.93	4.57	4.65	4.81	4.57	4.65	4.77	4.74	4.30	4.48	4.87	4.71	4.01	4.44	4.39	4.39	4.39
rno-miR-99a	8.04	7.74	7.96	7.96	7.61	8.16	7.96	7.96	8.29	7.80	8.16	8.16	8.29	8.29	8.16	8.47	8.16	7.88	7.96	8.04	8.16	8.16	7.96	7.74	7.96	7.96

Supplementary Table 2. Several miRNA-mRNA pairs involved in cell cycle regulation using IPA.

No.	ID	miRNA FC（2/3 vs 1/3）	Source	Confidence	ID	Symbol	mRNA FC（2/3 vs 1/3）	cell cycle associated pathway
1	rno-miR-25	1.54	TargetScan	High (predicted)	1379014_at	ARF1	-14.776	Integrin Signaling Paxillin Signaling
2	rno-miR-25	1.54	TargetScan	High (predicted)	1369658_at	CEBPA	-4.712	Acute Myeloid Leukemia Signaling Glucocorticoid Receptor Signaling Growth Hormone Signaling Role of Macrophages, Fibroblasts and Endothelial Cells in Rheumatoid Arthritis Tight Junction Signaling VDR/RXR Activation
3	rno-miR-25	1.54	TargetScan	High (predicted)	1393762_at	KAT2B	-2.829	AMPK Signaling Androgen Signaling Cell Cycle: G2/M DNA Damage Checkpoint Regulation Estrogen Receptor Signaling Glucocorticoid Receptor Signaling HMGB1 Signaling RAR Activation p53 Signaling
4	rno-miR-25	1.54	TargetScan	High (predicted)	1388250_at	NFIB	-2.696	Aryl Hydrocarbon Receptor Signaling

5	rno-miR-25	1.54	TargetScan	High (predicted)	1381213_at	NFIX	-7.799	Aryl Hydrocarbon Receptor Signaling
6	rno-miR-25	1.54	TargetScan	High (predicted)	1374593_at	PRKCE	-1.723	14-3-3-mediated Signaling Aldosterone Signaling in Epithelial Cells Amyloid Processing Androgen Signaling Apoptosis Signaling Axonal Guidance Signaling Breast Cancer Regulation by Stathmin1 CCR3 Signaling in Eosinophils CCR5 Signaling in Macrophages CREB Signaling in Neurons CXCR4 Signaling Calcium-induced T Lymphocyte Apoptosis Cholecystokinin/Gastrin-mediated Signaling Corticotropin Releasing Hormone Signaling Dopamine-DARPP32 Feedback in cAMP Signaling ERK/MAPK Signaling Endothelin-1 Signaling ErbB Signaling ErbB4 Signaling Erythropoietin Signaling Factors Promoting Cardiogenesis in Vertebrates Fc Epsilon RI Signaling Fcγ Receptor-mediated Phagocytosis in Macrophages and Monocytes G Beta Gamma Signaling G-Protein Coupled Receptor Signaling GNRH Signaling Gap Junction Signaling Glioma Signaling Growth Hormone Signaling Gαq Signaling HER-2 Signaling in Breast Cancer HGF Signaling Hepatic Cholestasis Huntington's Disease Signaling IL-12 Signaling and Production in Macrophages IL-3 Signaling IL-8 Signaling LPS-stimulated MAPK Signaling Leukocyte Extravasation Signaling Macropinocytosis Signaling Mechanisms of Viral Exit from Host Cells Melatonin Signaling Molecular Mechanisms of Cancer NF-κB Activation by Viruses NRF2-mediated Oxidative Stress Response Natural Killer Cell Signaling Neuregulin Signaling Neuropathic Pain Signaling In Dorsal Horn Neurons P2Y Purigenic Receptor Signaling Pathway Phospholipase C Signaling Production of Nitric Oxide and Reactive Oxygen Species in Macrophages Prolactin Signaling Protein Kinase A Signaling Pyridoxal 5'-phosphate Salvage Pathway RAR Activation Renin-Angiotensin Signaling Role of Macrophages, Fibroblasts and Endothelial Cells in Rheumatoid Arthritis Role of NFAT in Cardiac Hypertrophy Role of Pattern Recognition Receptors in Recognition of Bacteria and Viruses Salvage Pathways of Pyrimidine Ribonucleotides Sperm Motility Synaptic Long Term Depression Synaptic Long Term Potentiation Tec Kinase Signaling Thrombin Signaling Thrombopoietin Signaling Type II Diabetes Mellitus Signaling UVB-Induced MAPK Signaling UVC-Induced MAPK Signaling VDR/RXR Activation VEGF Family Ligand-Receptor Interactions Virus Entry via Endocytic Pathways Xenobiotic Metabolism Signaling fMLP Signaling in Neutrophils mTOR Signaling nNOS Signaling in Neurons p70S6K Signaling α-Adrenergic Signaling
7	rno-miR-25	1.54	Ingenuity Expert Findings,TargetScan	Experimentally	137011	PTEN	-1.552	3-phosphoinositide Biosynthesis 3-phosphoinositide Degradation B Cell Receptor Signaling D-myo-inositol (1,3,4)-trisphosphate Biosynthesis D-myo-inositol (1,4,5,6)-Tetrakisphosphate Biosynthesis D-myo-inositol

			Human	Observed,High (predicted)	2_at			(3,4,5,6)-tetrakisphosphate Biosynthesis D-myo-inositol-5-phosphate Metabolism Endometrial Cancer Signaling Epithelial Adherens Junction Signaling ErbB2-ErbB3 Signaling FAK Signaling Fcγ Receptor-mediated Phagocytosis in Macrophages and Monocytes Glioblastoma Multiforme Signaling Glioma Signaling Hereditary Breast Cancer Signaling Hypoxia Signaling in the Cardiovascular System IL-17A Signaling in Airway Cells ILK Signaling Insulin Receptor Signaling Integrin Signaling Melanoma Signaling Neuregulin Signaling Ovarian Cancer Signaling PI3K Signaling in B Lymphocytes PI3K/AKT Signaling PTEN Signaling Prostate Cancer Signaling Protein Kinase A Signaling RAR Activation Role of Tissue Factor in Cancer Sertoli Cell-Sertoli Cell Junction Signaling Small Cell Lung Cancer Signaling Superpathway of D-myo-inositol (1,4,5)-trisphosphate Metabolism Superpathway of Inositol Phosphate Compounds Tight Junction Signaling iCOS-iCOSL Signaling in T Helper Cells p53 Signaling
8	rno-miR-25	1.54	TargetScan	High (predicted)	1388215_at	PVRL1	-5.782	Epithelial Adherens Junction Signaling Sertoli Cell-Sertoli Cell Junction Signaling Tight Junction Signaling
9	rno-miR-25	1.54	TargetScan	High (predicted)	1369071_at	S1PR1	-3.034	Ceramide Signaling G-Protein Coupled Receptor Signaling Gαi Signaling Human Embryonic Stem Cell Pluripotency Sphingosine-1-phosphate Signaling cAMP-mediated signaling
10	rno-miR-25	1.54	TargetScan	High (predicted)	1392802_at	SMURF1	-2.237	BMP signaling pathway Protein Ubiquitination Pathway Regulation of the Epithelial-Mesenchymal Transition Pathway Role of Osteoblasts, Osteoclasts and Chondrocytes in Rheumatoid Arthritis TGF-β Signaling Tight Junction Signaling
11	rno-miR-25	1.54	TargetScan	High (predicted)	1391404_at	STAG2	-16.024	Mitotic Roles of Polo-Like Kinase
1	rno-miR-93/rno-miR-106b	1.9/1.3	TargetScan	High (predicted)	1383469_at	ALDH1A3	-2.104	Aryl Hydrocarbon Receptor Signaling Dopamine Degradation Ethanol Degradation II Ethanol Degradation IV Fatty Acid α-oxidation Histamine Degradation LPS/IL-1 Mediated Inhibition of RXR Function Noradrenaline and Adrenaline Degradation Oxidative Ethanol Degradation III Putrescine Degradation III RAR Activation Retinoate Biosynthesis I Serotonin Degradation Tryptophan Degradation X (Mammalian, via Tryptamine) Xenobiotic Metabolism Signaling
2	rno-miR-9	1.9/1.3	TarBase,TargetScan	Experimental	136	ARI	-1.784	DNA Methylation and Transcriptional Repression Signaling

	3/rno-miR-106b		Human,miRecords	ly Observed,High (predicted)	8837_at	D4B		
3	rno-miR-93/rno-miR-106b	1.9/1.3	miRecords	Experimentally Observed	1387611_at	BCL2	-1.590	Amyotrophic Lateral Sclerosis Signaling Apoptosis Signaling Ceramide Signaling Cytotoxic T Lymphocyte-mediated Apoptosis of Target Cells Death Receptor Signaling Docosahexaenoic Acid (DHA) Signaling Glucocorticoid Receptor Signaling Hepatic Fibrosis / Hepatic Stellate Cell Activation IL-15 Signaling IL-8 Signaling Induction of Apoptosis by HIV1 Interferon Signaling Melanocyte Development and Pigmentation Signaling Molecular Mechanisms of Cancer Myc Mediated Apoptosis Signaling Nur77 Signaling in T Lymphocytes OX40 Signaling Pathway Ovarian Cancer Signaling PEDF Signaling PI3K/AKT Signaling PTEN Signaling Pancreatic Adenocarcinoma Signaling Prostate Cancer Signaling Role of MAPK Signaling in the Pathogenesis of Influenza Role of Osteoblasts, Osteoclasts and Chondrocytes in Rheumatoid Arthritis Small Cell Lung Cancer Signaling TGF-β Signaling Type I Diabetes Mellitus Signaling VEGF Signaling p53 Signaling
4	rno-miR-93/rno-miR-106b	1.9/1.3	TargetScan	High (predicted)	1380287_at	CREB5	-23.092	ATM Signaling B Cell Receptor Signaling CREB Signaling in Neurons Calcium Signaling Circadian Rhythm Signaling Corticotropin Releasing Hormone Signaling Dendritic Cell Maturation Dopamine-DARPP32 Feedback in cAMP Signaling ERK/MAPK Signaling ERK5 Signaling Ephrin Receptor Signaling Estrogen-Dependent Breast Cancer Signaling FGF Signaling FLT3 Signaling in Hematopoietic Progenitor Cells G-Protein Coupled Receptor Signaling GNRH Signaling Gαs Signaling Huntington's Disease Signaling Hypoxia Signaling in the Cardiovascular System ILK Signaling Melanocyte Development and Pigmentation Signaling NGF Signaling Neurotrophin/TRK Signaling P2Y Purigenic Receptor Signaling Pathway Phospholipase C Signaling Prostate Cancer Signaling Protein Kinase A Signaling Role of IL-17F in Allergic Inflammatory Airway Diseases Role of Macrophages, Fibroblasts and Endothelial Cells in Rheumatoid Arthritis Synaptic Long Term Potentiation cAMP-mediated signaling p38 MAPK Signaling
5	rno-miR-93/rno-miR-106b	1.9/1.3	TargetScan	High (predicted)	1393762_at	KAT2B	-2.829	AMPK Signaling Androgen Signaling Cell Cycle: G2/M DNA Damage Checkpoint Regulation Estrogen Receptor Signaling Glucocorticoid Receptor Signaling HMGB1 Signaling RAR Activation p53 Signaling
6	rno-miR-93/rno-miR-106b	1.9/1.3	TargetScan	High (predicted)	1398174_at	MECP2	-2.111	DNA Methylation and Transcriptional Repression Signaling

7	rno-miR-93/rno-miR-106b	1.9/1.3	TargetScan	High (predicted)	1388250_at	NFIB	-2.696	Aryl Hydrocarbon Receptor Signaling
8	rno-miR-93/rno-miR-106b	1.9/1.3	TargetScan	High (predicted)	1369221_at	PPP2R2A	-1.745	AMPK Signaling Breast Cancer Regulation by Stathmin1 CDK5 Signaling CTLA4 Signaling in Cytotoxic T Lymphocytes Cardiac β-adrenergic Signaling Cell Cycle Regulation by BTG Family Proteins Ceramide Signaling Cyclins and Cell Cycle Regulation Dopamine Receptor Signaling Dopamine-DARPP32 Feedback in cAMP Signaling ERK/MAPK Signaling ILK Signaling Mitotic Roles of Polo-Like Kinase PI3K/AKT Signaling Production of Nitric Oxide and Reactive Oxygen Species in Macrophages Regulation of eIF4 and p70S6K Signaling Role of CHK Proteins in Cell Cycle Checkpoint Control Synaptic Long Term Depression Telomerase Signaling Tight Junction Signaling Wnt/β-catenin Signaling Xenobiotic Metabolism Signaling mTOR Signaling p70S6K Signaling
9	rno-miR-93/rno-miR-106b	1.9/1.3	Ingenuity Expert Findings,TargetScan Human	Experimentally Observed,High (predicted)	1370112_at	PTEN	-1.552	3-phosphoinositide Biosynthesis 3-phosphoinositide Degradation B Cell Receptor Signaling D-myo-inositol (1,3,4)-trisphosphate Biosynthesis D-myo-inositol (1,4,5,6)-Tetrakisphosphate Biosynthesis D-myo-inositol (3,4,5,6)-tetrakisphosphate Biosynthesis D-myo-inositol-5-phosphate Metabolism Endometrial Cancer Signaling Epithelial Adherens Junction Signaling ErbB2-ErbB3 Signaling FAK Signaling Fcγ Receptor-mediated Phagocytosis in Macrophages and Monocytes Glioblastoma Multiforme Signaling Glioma Signaling Hereditary Breast Cancer Signaling Hypoxia Signaling in the Cardiovascular System IL-17A Signaling in Airway Cells ILK Signaling Insulin Receptor Signaling Integrin Signaling Melanoma Signaling Neuregulin Signaling Ovarian Cancer Signaling PI3K Signaling in B Lymphocytes PI3K/AKT Signaling PTEN Signaling Prostate Cancer Signaling Protein Kinase A Signaling RAR Activation Role of Tissue Factor in Cancer Sertoli Cell-Sertoli Cell Junction Signaling Small Cell Lung Cancer Signaling Superpathway of D-myo-inositol (1,4,5)-trisphosphate Metabolism Superpathway of Inositol Phosphate Compounds Tight Junction Signaling iCOS-iCOSL Signaling in T Helper Cells p53 Signaling
10	rno-miR-93/rno-miR-106b	1.9/1.3	Ingenuity Expert Findings,TarBase,TargetScan Human,miRecords	High (predicted)	1388185_at	RB1	-1.674	Antiproliferative Role of TOB in T Cell Signaling Aryl Hydrocarbon Receptor Signaling Bladder Cancer Signaling Cell Cycle Regulation by BTG Family Proteins Cell Cycle: G1/S Checkpoint Regulation Chronic Myeloid Leukemia Signaling Cyclins and Cell Cycle Regulation Estrogen-mediated S-phase Entry Glioblastoma Multiforme Signaling Glioma Signaling Hereditary Breast Cancer Signaling Melanoma Signaling Molecular Mechanisms of Cancer Non-Small Cell Lung Cancer Signaling Ovarian Cancer Signaling Pancreatic Adenocarcinoma Signaling Prostate Cancer Signaling Regulation of Cellular Mechanics

								by Calpain Protease Role of BRCA1 in DNA Damage Response Role of Oct4 in Mammalian Embryonic Stem Cell Pluripotency Role of p14/p19ARF in Tumor Suppression Small Cell Lung Cancer Signaling Telomerase Signaling p53 Signaling
11	rno-miR-93/rno-miR-106b	1.9/1.3	TargetScan	High (predicted)	1377663_at	RND3	-1.819	Actin Nucleation by ARP-WASP Complex CXCR4 Signaling Cardiac Hypertrophy Signaling Cholecystokinin/Gastrin-mediated Signaling Colorectal Cancer Metastasis Signaling Germ Cell-Sertoli Cell Junction Signaling Glioblastoma Multiforme Signaling Glioma Invasiveness Signaling Gαq Signaling HMGB1 Signaling IL-8 Signaling ILK Signaling Integrin Signaling Molecular Mechanisms of Cancer Phospholipase C Signaling Production of Nitric Oxide and Reactive Oxygen Species in Macrophages Regulation of Actin-based Motility by Rho RhoA Signaling RhoGDI Signaling Semaphorin Signaling in Neurons Signaling by Rho Family GTPases Sphingosine-1-phosphate Signaling Tec Kinase Signaling Thrombin Signaling mTOR Signaling
12	rno-miR-93/rno-miR-106b	1.9/1.3	TargetScan	Experimentally Observed,High (predicted)	1369071_at	S1PR1	-3.034	Ceramide Signaling G-Protein Coupled Receptor Signaling Gαi Signaling Human Embryonic Stem Cell Pluripotency Sphingosine-1-phosphate Signaling cAMP-mediated signaling
13	rno-miR-93/rno-miR-106b	1.9/1.3	TargetScan	High (predicted)	1392802_at	SMURF1	-2.237	BMP signaling pathway Protein Ubiquitination Pathway Regulation of the Epithelial-Mesenchymal Transition Pathway Role of Osteoblasts, Osteoclasts and Chondrocytes in Rheumatoid Arthritis TGF-β Signaling Tight Junction Signaling
14	rno-miR-93/rno-miR-106b	1.9/1.3	TargetScan	High (predicted)	1375364_at	STK11	-14.727	14-3-3-mediated Signaling AMPK Signaling mTOR Signaling
15	rno-miR-93/rno-miR-106b	1.9/1.3	Ingenuity Expert Findings,TarBase,TargetScan Human	Experimentally Observed,High (predicted)	1369653_at	TGFBR2	-1.679	Antiproliferative Role of TOB in T Cell Signaling Cardiac Hypertrophy Signaling Chronic Myeloid Leukemia Signaling Colorectal Cancer Metastasis Signaling Epithelial Adherens Junction Signaling Factors Promoting Cardiogenesis in Vertebrates Germ Cell-Sertoli Cell Junction Signaling Glucocorticoid Receptor Signaling Hepatic Fibrosis / Hepatic Stellate Cell Activation Human Embryonic Stem Cell Pluripotency Inhibition of Angiogenesis by TSP1 Molecular Mechanisms of Cancer NF-κB Signaling PPARα/RXRα Activation PTEN Signaling Pancreatic Adenocarcinoma Signaling Protein Kinase A Signaling Regulation of IL-2 Expression in Activated and Anergic T Lymphocytes Regulation of the Epithelial-Mesenchymal Transition Pathway Role of NFAT in Cardiac Hypertrophy T Helper Cell Differentiation TGF-β Signaling Tight Junction Signaling

								Wnt/β-catenin Signaling p38 MAPK Signaling
16	rno-miR-93/rno-miR-106b	1.9/1.3	TargetScan	High (predicted)	1381167_at	TIAM1	-4.953	Actin Cytoskeleton Signaling Rac Signaling Tight Junction Signaling

Supplementary Methods

miRNA expression profiling using microarrays

Three samples for each type of surgery at each time point were used for microarray analysis. Total RNA was extracted and purified using the mirVana™ miRNA Isolation Kit (Ambion) according to the manufacturer's instructions and was confirmed for the RIN number to inspect RNA integration using an Agilent Bioanalyzer 2100 (Agilent Technologies). The molecular miRNA within the total RNA was labeled by the miRNA Complete Labeling and Hyb Kit (Agilent Technologies) followed the manufacturer's instructions for labeling. Each slide was hybridized with 100 ng Cy3-labeled RNA using the miRNA Complete Labeling and Hyb Kit in hybridization Oven (Agilent Technologies) at 55℃, 20rpm for 20 hours according to the manufacturer's instructions for hybridization. After hybridization, the slides were washed in staining dishes (Thermo) with the Gene Expression Wash Buffer Kit (Agilent Technologies). Slides were scanned using an Agilent Microarray Scanner (Agilent Technologies), and Feature Extraction software 10.7 (Agilent Technologies) was used with the default settings. Raw data were normalized against the Quantile algorithm, Gene Spring Software 11.0 (Agilent Technologies).

mRNA expression profiling using microarrays

Total mRNA was amplified, labeled and purified using the GeneChip 3'IVT Express Kit (Affymetrix) followed the manufacturer's instructions to obtain biotin labeled cRNA. Array hybridization and wash were performed using the GeneChip® Hybridization, Wash and Stain Kit (Affymetrix) in a Hybridization Oven 645 (Affymetrix) and with a Fluidics Station 450 (Affymetrix) according to the manufacturer's instructions. Slides were scanned using the GeneChip® Scanner 3000 (Affymetrix) and Command Console Software 3.1 (Affymetrix) with default settings. Raw data were normalized by MAS 5.0 algorithm in Gene Spring Software 11.0 (Agilent Technologies).

Quantitative reverse transcription-polymerase chain reaction (qRT-PCR)

cDNA was synthesized from 10 ng of each RNA sample using the miScript II Reverse

Transcription Kit (Qiagen). PCR was performed using SYBR Green microRNA Assays. Rno-U6 snRNA served as the endogenous control. miRNA-specific primers were synthesized using Generay. The qRT-PCR was carried out on Applied Biosystems 7900HT Real-Time PCR System (AppliedBiosystem) using the Power SYBRH Green PCR Master Mix kit (Roche). The PCR conditions included an initial denaturation at 95℃ for 10 min, a two-step cycling conditions: 15 s denaturation at 95° C and annealing/extension at 60° C for 30 s, cycled 40 times. PCR reactions were performed in triplicate. The expression levels were calculated using the $\Delta\Delta$Ct method.

Immunohistochemical staining and western blotting analyses

For immunohistochemical staining, bromodeoxyuridine (BrdU) was injected intraperitoneally (50 mg/kg) 2 hours prior to liver harvest to detect cells undergoing DNA synthsis. Harvested livers were embedded into paraffin blocks after fixation. Four μm paraffin sections were prepared on slides for BrdU, and Ki67 staining. Briefly, the slides were first deparaffinized by sequential treatment with xylene, 100% ethanol, 90% ethanol, 70% ethanol, 50% ethanol, distilled water, and PBS. To reveal BrdU labeling, the slides were then processed by using a BrdU staining kit (Invitrogen) according to the manufacturer’s instructions. For Ki67 staining, the slides were incubated for 20 minutes in 1% H2O2 and washed 3 times with PBS. The slides were then blocked with a 1:2 dilution of fetal bovine serum for 1 hour. Tissue sections were treated with rabbit monoclonal Ki67 antibody at 1:200 with PBS containing blocking solution for 2 hours at 37℃ in a humidified chamber. After incubation, each slide was washed 3 times with PBS. A 1:300 dilution of horseradish peroxidase-labeled goat anti-rabbit antibody was incubated on the slides for 2 hours at 37℃ in a humidified chamber. The slides were then washed 3 times with PBS. The results were visualized via a reaction with diaminobenzidine (DAB, 3, 30-diaminobenzidine tetrahydrochloride) and counterstained with hematoxylin (Sigma). The number of cells that stained positively for BrdU per low-power field were quantified and expressed as the mean ± the SEM for each group.

For western blotting analysis, cultured cells were collected in RIPA lysis buffer (50 mM Tris–HCl, 150 mM NaCl, 1% Triton X-100, 1% sodium deoxycholate, 0.1% SDS, 1 mM PMSF) with 10 mg/ml protease inhibitor cocktail (Sigma). The extracted proteins were separated by SDS-PAGE and were transferred onto PVDF membranes. After being blocked by TBS-T buffer containing 5% non-fat milk powder for 2 hours, the membranes were immunoblotted using a primary antibody against RB1 (1:1500, Sigma) and KAT2B (1:1000, BAioworld). Immunoreactive bands on the blots were

visualized with an enhanced chemiluminescence reagent ECL kit (Beit Haemek).

Cell cycle and apoptosis analysis using flow cytometry

Two rat cell lines (BRL, normal hepatic cell line; RH-35, hepatoma cell line) were purchased from the Institute of Biochemistry and Cell Biology, Chinese Academy of Sciences (Shanghai, China) and maintained in Dulbecco's modified Eagle's medium (DMEM, Gibco) supplemented with 10% fetal bovine serum (Gibco) in a 37 ℃ incubator with 5% CO2. Cells were plated at a density of 3×105 cells per well of a 6-well Primaria plate (BD Bioscience). Twenty-four hours after seeding, the cells were transfected with control or miRNA mimics using HiPerFect Transfection Reagent (Qiagen) and the cells were collected 48 hours after transfection. For cell cycle analysis, 10^6 cells were re-suspended in 300 μl of PBS, and 700 μl ice cold ethanol to fix the cells overnight at -20 ℃. Subsequently, the cells were washed twice with PBS and were then stained with 0.5 ml of DNA Prep Stain from the Coulter DNA-Prep Reagents kit (Beckman Coulter) at room temperature for 30 minutes in the dark followed by flow cytometry (BD Biosciences). The data were analyzed to calculate the percentage of the cell population in each phase using ModFit LT software. For apoptosis assay, cells were evaluated by using FITC-conjugated Annexin-V and Propidium Iodide (PI) kit according to the manufacturer's instructions and were analyzed using a flow cytometer and FlowJo software. APC-AnnexinV and 7-AAD staining were performed according to the manufacturer's instructions. Samples were analyzed using flow cytometry.

Adeno-associated virus (AAV) 9 preparation

miRNA precursor sequences of miR-106b~25 and sponges for these miRNAs were synthesized and confirmed by sequencing. The miRNA precursor and sponges were inserted in the plasmid pAOV-CMV-mCherry and pAOV-CMV-GFP, respectively (Neuronbiotech, Shanghai, China). As a negative control, an adenoviral vector expressing green fluorescent protein (GFP) or mCherry was constructed. AAV was produced by the transfection of 293T cells with 3 plasmids: an AAV vector (expressing the cluster, sponges, and their respective controls), an AAV helper plasmid (pAAV Helper) and an AAV Rep/Cap expression plasmid. At 72 h after transfection, the cells were collected and lysed using a freeze-thaw protocol. The viruses were purified using cesium chloride density gradient dialysis. All vector preparations were tittered by quantitative PCR using Taq-Man technology. The purity of the vectors was assessed by 4–12% SDS-acrylamide gel electrophoresis and silver

staining. The titer of the viruses was then determined by quantitative polymerase chain reaction (qPCR): 2×10^{12}–3×10^{12} infectious units per ml. The sequence of the miR-106b-25 was
catcactgcagcgtatgtggagatgaggcgagaggcccgggccagtaaggatgccacctacacttctgcccggaccctg
ctggccattctccgactttctactgctctggtgagtgctgggtccctgtctgctagaaggctgacccttgcctgccacctcacc
taatgtccttcaagctgccttcttccctcccaccagccctgctgggactaaagtgctgacagtgcagatagtggtcctctctgt
gctaccgcactgtgggtacttgctgctccagcagggcacatgcaacacccacggagggaaaggctgcttgaatccacga
gggggttggaaggacttgagttcctgatgccctagatatcttagaagtcttccttctgtcctcccctcttggacctttagtcatgg
gggctccaaagtgctgttcgtgcaggtagtgcattgcctgacctactgctgagctagcacttcccgagcccccaggacaca
gcctctgacagtggccctagctgagcaccccaggctccacttgtcacgatggggagcctagtggagttcagaagggtctg
gtctccctcacaggacggcaggacacccaccgggactggccagtgttgagaggcggagacacgggcaattgctggacg
ctgccctgggcattgcacttgtctcggtctgacagtgccggcccaacactgcagatgtgggggcagggaagaacccact
tttgttctatgagtaggcgaatgtggcttgcccgggtttgaagccagagcagagatccttggtttcacccctcctcccctcctat
ccctatttccccacgccttccccttaggctcgccttcggatggtagatattgtggagaaggaagatgtgaatgaagcc. The sequence of the sponges of miR-106b-25 was
atctgcactgcgaagcactttagcagatctgcactgcgaagcactttagcagctacctgcactcaagcactttggcagtcag
accgagcacagtgcaatggcagctacctgcactcaagcactttggcagtcagaccgagcacagtgcaatggcagatctgc
actgcgaagcactttagcagatctgcactgcgaagcactttagcagctacctgcactcaagcactttggcagtcagaccga
gcacagtgcaatggcagctacctgcactcaagcactttggcagtcagaccgagcacagtgcaatggcagatctgcactgc
gaagcactttagcagatctgcactgcgaagcactttagcagctacctgcactcaagcactttggcagtcagaccgagcaca
gtgcaatggcagctacctgcactcaagcactttggcagtcagaccgagcacagtgcaatggcag. 2×1012 viral particles of adeno-associated virus (AAV) 9-miR-cluster, AAV9-miR-sponge, and their respective controls were separately injected into rats by way of tail vein.

Blood laboratory tests

Blood samples were obtained at animal sacrifice from the inferior vena cava in rats that had been injected with the AAV9 virus. Serum alanine aminotransferase (ALT), aspartate aminotransferase (AST), and the serum total bilirubin (T.Bil) levels were measured using standard laboratory methods.

Luciferase reporter assay

A total of 5×10^4 293T cells per well of a 24-well plate were seeded 1 day prior to transfection. Transfections were performed with Lipofectamine 2000 (Invitrogen) according to the manufacturer’s instructions. For the luciferase assays, 293T cells were cotransfected with 10 nmol/L miRNA mimics or with the control or different pMIR-constructs. Transfected cells were assayed at 48 hours posttransfection and

firefly and Renilla luciferase activities were measured using the Dual-Luciferase Reporter Assay System (Promega). The relative luciferase activity was calculated as the ratio between the Renilla and firefly luciferase activities.
